# Identification of STAU1 as a regulator of HBV replication by TurboID-based proximity labeling

**DOI:** 10.1016/j.isci.2022.104416

**Published:** 2022-05-18

**Authors:** Xia-Fei Wei, Shu-Ying Fan, Yu-Wei Wang, Shan Li, Shao-Yuan Long, Chun-Yang Gan, Jie Li, Yu-Xue Sun, Lin Guo, Pei-Yun Wang, Xue Yang, Jin-Lan Wang, Jing Cui, Wen-Lu Zhang, Ai-Long Huang, Jie-Li Hu

**Affiliations:** 1 Key Laboratory of Molecular Biology on Infectious Diseases, Ministry of Education, Chongqing Medical University, Chongqing, China; 2 Institute for Hepatology, National Clinical Research Center for Infectious Disease, Shenzhen Third People’s Hospital, Southern University of Science and Technology, Shenzhen, China; 3 Department of Laboratory Medicine, Chongqing Hospital of Traditional Chinese Medicine, Chongqing, China

**Keywords:** Molecular biology, Virology

## Abstract

The core promoter (CP) of hepatitis B virus (HBV) is critical for HBV replication by controlling the transcription of pregenomic RNA (pgRNA). Host factors regulating the activity of the CP can be identified by different methods. Biotin-based proximity labeling, a powerful method with the capability to capture weak or dynamic interactions, has not yet been used to map proteins interacting with the CP. Here, we established a strategy, based on the newly evolved promiscuous enzyme TurboID, for interrogating host factors regulating the activity of HBV CP. Using this strategy, we identified STAU1 as an important factor involved in the regulation of HBV CP. Mechanistically, STAU1 indirectly binds to CP mediated by TARDBP, and recruits the SAGA transcription coactivator complex to the CP to upregulate its activity. Moreover, STAU1 binds to HBx and enhances the level of HBx by stabilizing it in a ubiquitin-independent manner.

## Highlights

• HBV core promoter binding factors were interrogated by a proximity labeling method
• STAU1 enhances HBV transcription and replication
• STAU1 indirectly binds to core promoter via TARDBP and recruits SAGA complex
• STAU1 binds to HBx and increases its stability

## Graphical abstract

**Figure:**
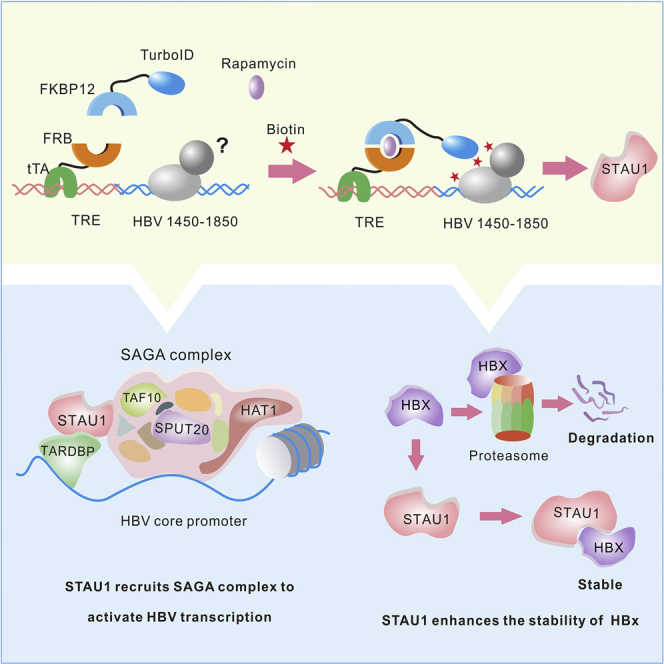

## Introduction

Hepatitis B virus (HBV) chronically infects approximately 257 million individuals worldwide and leads to 700,000 deaths per year owing to severe liver diseases, including acute liver failure, liver fibrosis, liver cirrhosis, and hepatocellular carcinoma (Hu et al., 2019; Polaris Observatory, 2018; Seeger and Mason, 2015). HBV is a hepatotropic and small DNA virus with a partially double-stranded relaxed circular DNA (rcDNA) genome (Tong and Revill, 2016). After entering into a hepatocyte, the HBV rcDNA can be converted into covalently closed circular DNA (cccDNA) in the nucleus that serves as the template for the transcription of all HBV RNAs, including the pregenomic RNA (pgRNA), pre-core (preC), preS1, S, and X RNA (Seeger and Mason, 2015; Venkatakrishnan and Zlotnick, 2016). Moreover, the transcription of cccDNA is controlled by two enhancers (Enh I and Enh II) and four promoters (core, preS1, S and x promoters) that interact with various host transcription factors (Quasdorff and Protzer, 2010; Yuh et al., 1992).

The core promoter (CP) controls the production of pgRNA, which serves as the mRNA for the expression of polymerase and core protein as well as the template of reverse transcription. The CP is therefore critical for the replication of HBV (Quarleri, 2014; Quasdorff and Protzer, 2010). Additionally, the activity of CP was reported to be regulated by nuclear receptors (NRs), liver-enriched factors and ubiquitous transcription factors, such as HNF4α, HNF1α, C/EBP, FTF, PPARα, RXRα, TR2, TR4, SP1, ZHX2, PROX1, SOX7, Slug, SOX9, PGC-1α, and so on (Mohd-Ismail et al., 2019; Turton et al., 2020). The discovery of these regulators led to a deeper understanding of how HBV exploits host factors to complete its life cycle and provided potential targets for the development of anti-HBV agents.

Moreover, different strategies have been used for the discovery of host factors regulating the activity of the HBV CP. For instance, many NRs bound to the CP were identified through binding-sequence guided speculation. Notably, the presence of DNA motifs in the CP, which are similar to those binding to specific NRs, provided initial clues. These clues, in turn, led to the identification of EF-C (David et al., 1995; Garcia et al., 1993), HNF-4 (Garcia et al., 1993; Guo et al., 1993), RXRα (Garcia et al., 1993; Huan and Siddiqui, 1992), COUP-TF (Garcia et al., 1993), TR4 (Lin et al., 2003), TR2(Lin et al., 2008), and STAT3 (Waris and Siddiqui, 2002), as CP or enhancer regulators. In addition, a yeast one-hybrid screening system was used to identify transcription-related factors, which led to the discovery of HLF, FTF, and E4BP4 that might bind to Enh II (Ishida et al., 2000). Moreover, ^32^P-labeled oligonucleotides from the HBV CP or enhancer were used as probes to screen binding proteins from a cDNA expression library. As a result, E2BP was found to be associated with HBV transcription (Tay et al., 1992). Other strategies, including high-throughput RNAi screening and transcriptome microarray analysis, have also been successfully used to identify HBV transcription-related factors (Ko et al., 2017; Nishitsuji et al., 2018; Sun et al., 2017). The fact that different factors have been identified through different strategies emphasizes the complementary effect of different methods, and implies that novel factors might be discovered through new approaches.

Notably, biotin-based proximity labeling (PL) is a recently developed and powerful approach that complements the classic Affinity Purification/Mass Spectrometry (AP/MS)-based interactome mapping (Qin et al., 2021). The key factors in this method are promiscuous enzymes that can covalently label biotin derivatives to proteins in the near vicinity (Trinkle-Mulcahy, 2019). The biotin-labeled proteins can then be readily captured by streptavidin beads for further MS analysis. Moreover, weak or dynamic interactions that can be lost in standard AP approaches can be captured through this method (Trinkle-Mulcahy, 2019). The tight association between biotin and streptavidin also allows high-stringency protein extraction methods that help minimize background contaminants. APEX2 and BioID are the commonly used enzymes in PL (Kim et al., 2016; Rees et al., 2015; Rhee et al., 2013; Zhou et al., 2019). APEX2 offers rapid labeling kinetics but utilizes H_2_O_2_ to promote the reaction, which may be toxic to the living cells (Lam et al., 2015; Zhou et al., 2019). On the one hand, BioID-based protein biotinylation is non-toxin and simple, but is associated with slow kinetics, The method takes at least 18-24 h of biotin labeling to produce sufficient biotinylated proteins for MS analysis (Chojnowski et al., 2018; Kim et al., 2016; Liu et al., 2018). Furthermore, an *Escherichia coli* (*E. coli*) biotin ligase (BirA) mutant, TurboID was recently generated (Branon et al., 2018) through yeast display directed evolution. TurboID combines the efficient kinetics of APEX2 with the non-toxicity of BioID, hence exhibiting high catalytic efficiency and labeling activity in PL applications (Doerr, 2018; May et al., 2020). These features have, in turn, resulted in the successful application of TurboID-based PL in mammalian cells (Branon et al., 2018; Chua et al., 2021; Bozal-Basterra et al., 2020), plants (Arora et al., 2020; Mair et al., 2019; Zhang et al., 2019), yeast (Branon et al., 2018; Larochelle et al., 2019), worm (Branon et al., 2018), fly (Branon et al., 2018), and so on. However, this method has not been extensively used in the discovery of host factors interacting with viruses.

The present study developed a TurboID-based PL approach to identify host factors that regulated the activity of the HBV CP. Our results revealed a number of proteins, some of which are known host factors involved in the replication of HBV. Interestingly, a majority of these proteins had not been reported previously as HBV transcription-related factors. Further analysis of these candidate genes identified STAU1 that indirectly interacts with the HBV CP. Moreover, the study assessed the mechanisms through which STAU1 affected the replication of HBV.

## Results

### Establishment of a PL system for identifying pgRNA-transcription related host factors

**Figure 1:**
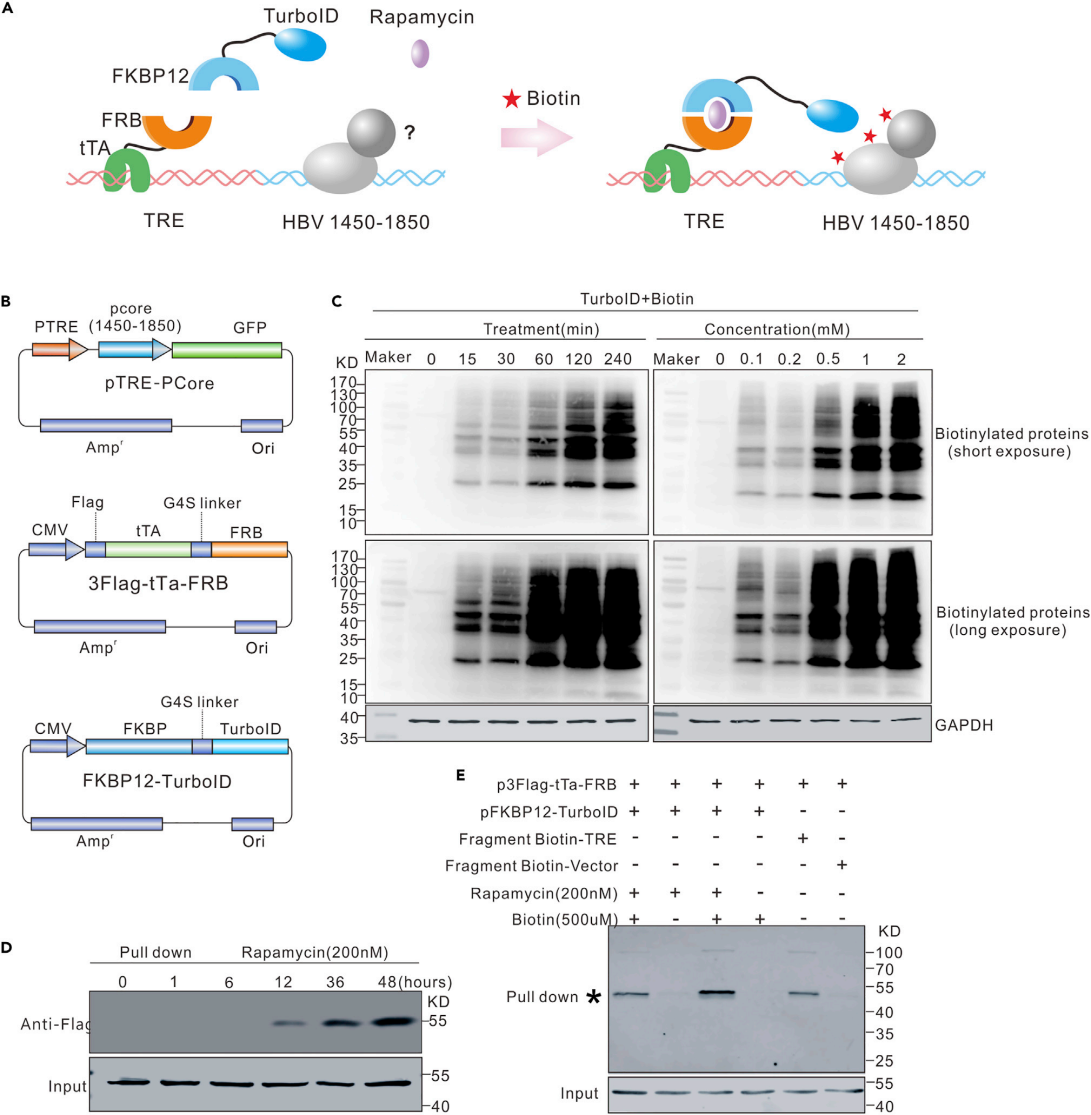
Establishment of a proximity labeling system for identifying transcription-related host factors (A) Strategy of the screening system for mapping transcription-related host factors of HBV core promoter/enhancer. (B) Structure of plasmids pTRE-PCore, 3Flag-tTa-FRB, and FKBP12-TurboID. (C) Optimization of biotin treatment. Plasmid FKBP12-TurboID was transfected into HepG2 cells. Effects of different biotin treatment conditions on the efficiency of biotinylated labeling of intracellular protein were analyzed 48 h post-transfection by Western blot. (D) Optimization of rapamycin treatment. Plasmids 3Flag-tTa-FRB and FKBP12-TurboID were co-transfected into HepG2 cells. Effects of different rapamycin-treatment periods on the labeling efficiency were assayed by pull-down experiments. (E) Evaluation of the efficacy of the proximity labeling system. Different plasmids or DNA fragments were transfected into HepG2 cells with the indicated combinations, and pull-down and Western blot were conducted to evaluate the labeling efficiency.

To identify host factors involved in the transcription of pgRNA, the study established a PL system based on TurboID. The system shown in Figures 1A and 1B included two parts. One was a DNA fragment containing tetracycline response elements (TRE), placed next to the HBV CP /enhancer sequence (HBV 1,450–1,850). In the presence of rapamycin, TRE can recruit the tetracycline transcriptional activating protein (tTA), fused with FRP (Banaszynski et al., 2005; Choi et al., 1996; Yang et al., 2013) that can further recruit FKBP12 linked to TurbolD (Baron and Bujard, 2000; Furth et al., 1994; Gossen and Bujard, 1992). On the other hand, the second part of the system, tTA-FRP plus FKBP12-TurbolD, consisting of biotinylated factors binding to the CP /enhancer adjacent to TRE. The biotinylated factors could then be isolated and characterized through mass spectrometry. The control samples were considered to be those without rapamycin treatment; therefore, FKBP12-TurbolD could not be recruited to the TRE.

In addition, to optimize the concentration and incubation time of exogenous biotin used in the system, we tested the biotinylated efficiency of proteins in HepG2 cells expressing FKBP12-TurboID. Based on the results, the levels of biotinylated proteins gradually increased with an increase in the incubation time and concentration of biotin (Figure 1C). Consequently, the study used 0.5 mM biotin and a 1-h incubation time, in the subsequent experiments. Moreover, the 3flag-tTa-FRB and plasmid FKBP12-TurboID plasmids were co-transfected into HepG2 cells, after which the cells were treated with rapamycin (200 nM) for different times, up to 48 h. This was done to optimize the concentration of rapamycin as well as the time of incubation. After the treatment with biotin (0.5 mM) for 1 h, the biotinylated proteins were pulled down using streptavidin beads and then analyzed through Western blot with Anti-Flag antibody. The results showed that the levels of biotinylated 3flag-tTa-FRB increased with an increase in the incubation time, for rapamycin. Notably, the increase was apparent after 12 h and reached a peak after 36 h (Figure 1D). Therefore, the study chose 200 nM rapamycin and treatment for 12 h, in the subsequent experiments.

To further evaluate the system, HepG2 cells were transfected with different combinations of pTER-PCORE, p3flag-tTa-FRB, pFKBP12-TurboID, biotinylated TRE fragment, or biotinylated control fragment (Figure 1E). The pull-down experiments with streptavidin beads and western blotting showed that the biotinylation of 3flag-tTa-FRB was regulated by both exogenous biotin and rapamycin. In addition, 3flag-tTa-FRB could be pulled down by the biotinylated TRE sequence but not the control sequence. These results therefore indicated that 3flag-tTa-FRB was biotinylated by FKBP12-TurboID mainly through the added biotin. The results also suggested that 3flag-tTa-FRB could bind to the TRE sequence.

### Identification of host factors binding to HBV CP /enhancer

**Figure 2:**
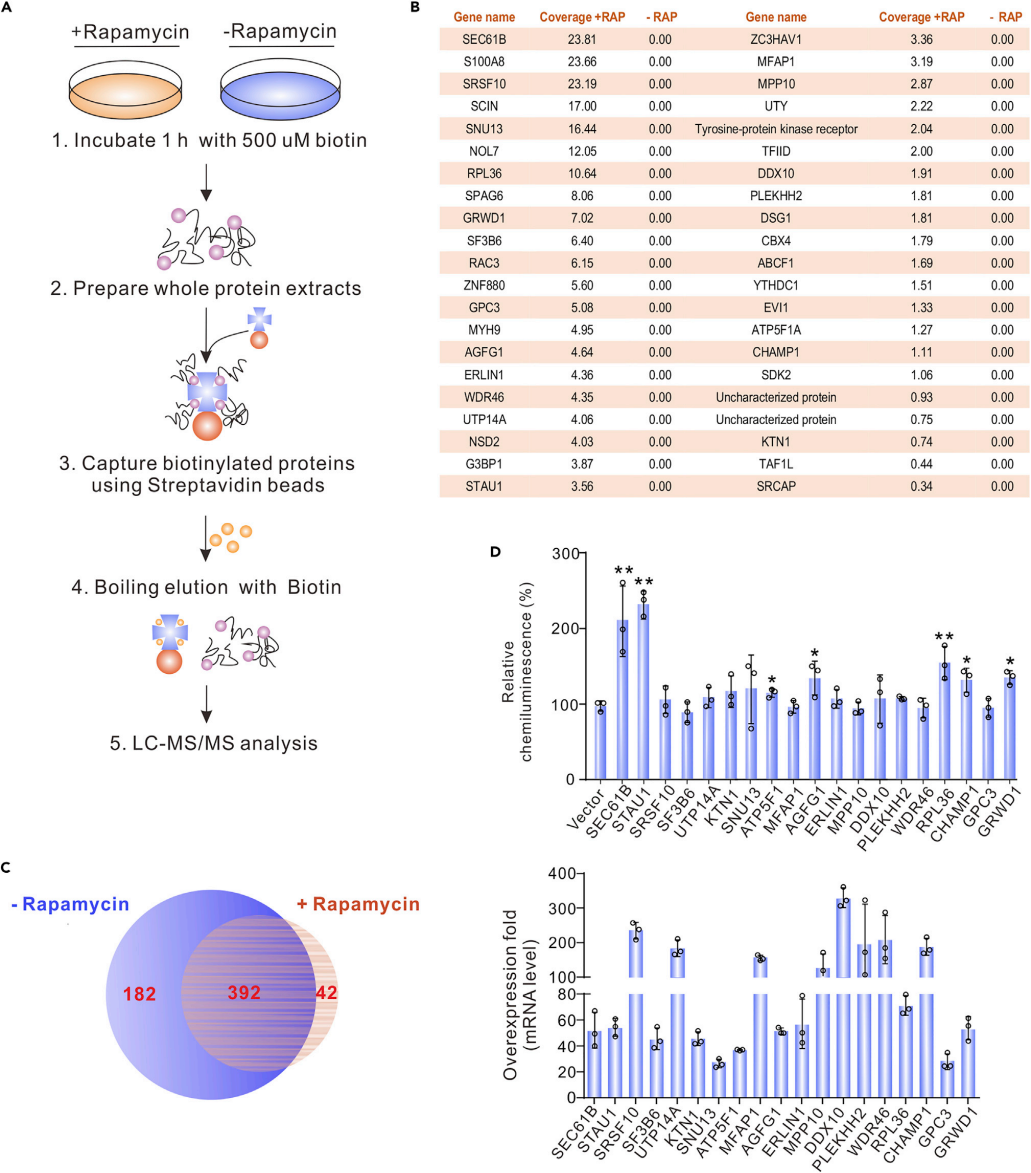
Identification of host factors binding to the HBV core promoter (A–C) (A) The flow-chart for screening transcription-related factors of the HBV core promoter. The results of LC-MS were shown in (B and C). (D) Effects of the 19 candidate genes on the activity of HBV core promoter. Expression plasmids of the candidate genes and plasmid pCH9-PCore-Rluc were co-transfected into HepG2 cells. The mRNA level of each gene and Renilla luciferase activity were detected. The data are presented as mean ± SD of three independent experiments. Significance was tested with an unpaired *t* test. ∗*p* < 0.05, ∗∗*p* < 0.01.

To identify host factors binding to the CP /enhancer, we co-transfected plasmids pTRE-PCORE, p3flag-tTa-FRB and pFKBP12-TurboID into HepG2 cells. The cells were treated with 200 nM rapamycin for 12 h or not (as a control), as shown in Figure 2A. Biotin (0.5 mM) was added into the culture medium to initiate labeling 1 h before lysis. Biotinylated proteins were enriched from the lysates using streptavidin beads, followed by liquid chromatography-mass spectrometry (LC-MS) analysis. Among the 434 proteins identified in the rapamycin-treated sample, 42 proteins were not present in the control (574 proteins) (Figures 2B and 2C; Table S4). We tried to clone all the 42 genes but succeeded with 19 genes. These constructs were co-transfected into HepG2 cells with a core-promoter-driving reporter plasmid for a second round of screening. As shown in Figure 2D, overexpression of SEC61B, STAU1, ATP5F1, RPL36, CHAMP1, and GRWD1 increased the renilla luciferase signal. Furthermore, the 19 constructs were co-transfected into HepG2 cells with pHBV1.3 and the replication of HBV DNA was analyzed by Southern blotting. In line with the reporter assays, the five genes (SEC61B, STAU1, RPL36, CHAMP1, and GRWD1) enhanced the level of intracellular HBV DNA (Figure S1A), and repeated assays revealed that STAU1 increases HBV replication the most (Figures S1B–S1E). Thus, a further study was mainly focused on STAU1.

### STAU1 promotes HBV replication by enhancing the transcription of HBV RNA

**Figure 3:**
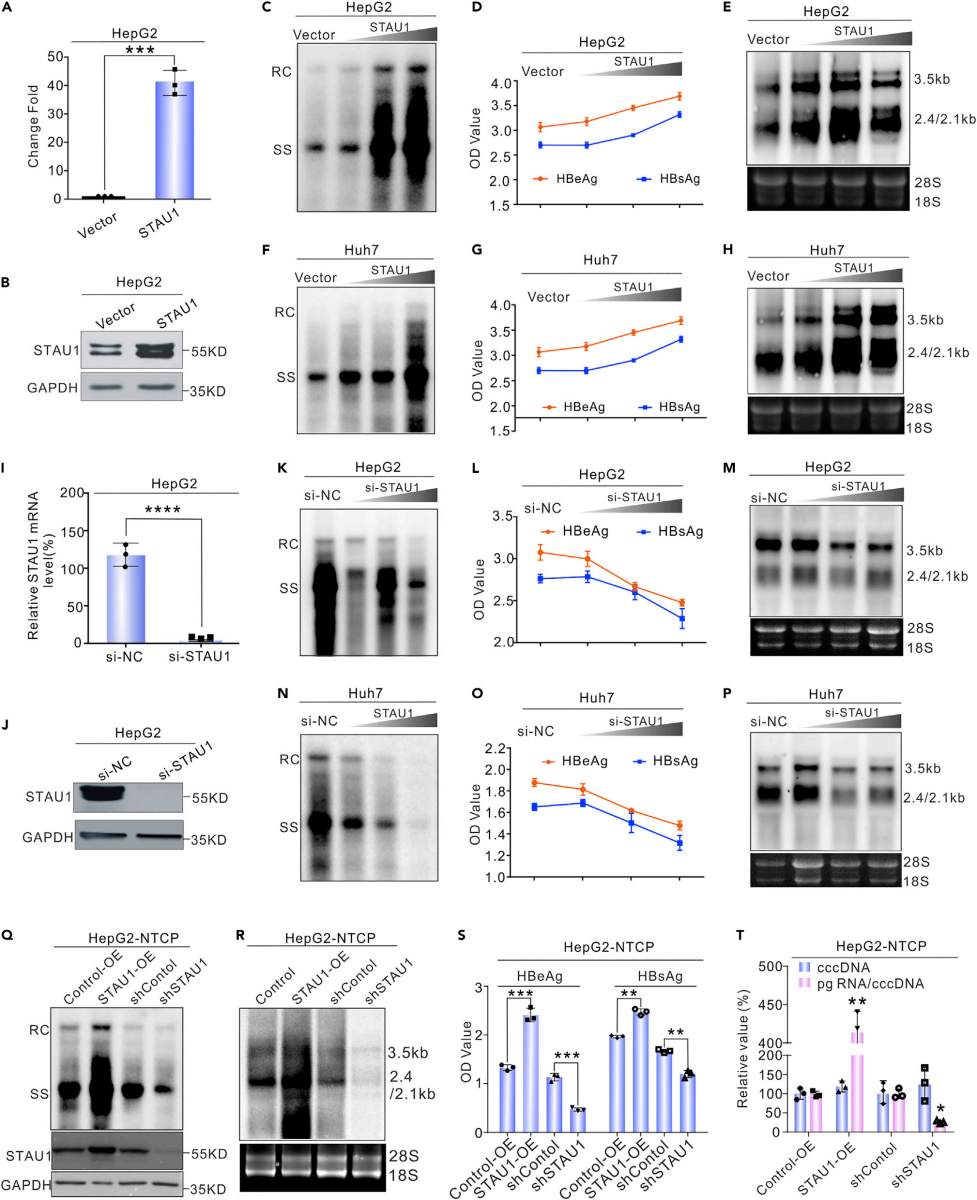
STAU1 enhances HBV replication in cultured cells (A and B) Validation of STAU1 overexpression in HepG2 cells. The data are presented as mean ± SD. ∗∗∗*p* < 0.001. (C–E) STAU1 overexpression increased HBV replication in HepG2 cells. Different amounts of pCH9-STAU1 (0, 0.2, 0.5, and 1 μg plus vector) and HBV1.3 were co-transfected into HepG2 cells. The intracellular HBV DNA, HBV RNA, and secreted HBsAg and HBeAg were detected 5 days post-transfection. (F–H) STAU1 overexpression enhanced HBV replication in Huh7 cells. (I and J) Knockdown efficiency of si-STAU1 evaluated by RT-qPCR and Western blot. The data are presented as mean ± SD. ∗∗∗∗*p* < 0.0001. (K–P) STAU1 knockdown suppressed HBV replication in HepG2 and Huh7 cells. Different amounts of si-STAU1 (0, 20, 50, and 100 pmol plus control siRNA) were co-transfected with HBV1.3 into HepG2 and Huh7 cells. The intracellular HBV DNA, HBV RNA, and secreted HBsAg and HBeAg were assayed. (Q–S) STAU1 increased HBV replication in the HepG2-NTCP cell model. Lentiviruses expressing STAU1 and shRNA against STAU1 and control lentivirus (Control-OE and shControl) were used to infect HepG2-NTCP cells, respectively. After blasticidin selection for one week, the surviving cells (pooled) were expanded for the infection experiments. After HBV infection, the cells were maintained for another 5 days and then intracellular HBV DNA, HBV RNA, and secreted HBsAg and HBeAg were assayed by Southern blot, Northern blot, and ELISA, respectively. (T) STAU1 did not affect cccDNA level in the HepG2-NTCP cell model. cccDNA and total RNA were extracted from the STAU1-OE, shSTAU1, and control cells that were infected with HBV and analyzed by qPCR. The data are presented as mean ± SD and analyzed with an unpaired *t* test. ∗*p* < 0.05, ∗∗*p* < 0.01, ∗∗∗*p* < 0.001.

To analyze the effect of STAU1 overexpression on HBV replication, we co-transfected STAU1-expressing plasmid with pHBV1.3 into cells. The overexpression of STAU1 was verified by RT-PCR and Western blot (Figures 3A and 3B). Southern blotting showed that intracellular HBV DNA levels increased with an increase in the amount of STAU1-expressing plasmid in both HepG2 and Huh7 cells (Figures 3C and 3F). The overexpression of STAU1 also increased HBsAg and HBeAg levels in the culture medium in a dose-dependent manner (Figures 3D and 3G). Northern blotting revealed that HBV RNAs were enhanced by STAU1 overexpression (Figures 3E and 3H).

To evaluate the effect of STAU1 knockdown on HBV replication, we synthesized siRNA modified with cholesterol against STAU1. The knockdown efficiency of the siRNA was verified by RT-qPCR and Western blot (Figures 3I and 3J). Knockdown of STAU1 inhibited the levels of HBV DNA, HBsAg, HBeAg, and HBV RNA in both HepG2 and Huh7 cells, respectively (Figures 3K–3P).

To further confirm the effects of STAU1, we constructed stable cell lines with STAU1 overexpression and knockdown based on HepG2-NTCP cells. These cell lines were infected with HBV and replication markers were analyzed. As shown in Figures 3Q–3S, HBV replication was significantly increased in the STAU1-overexpressed cell line as compared with the control cell line. On the other hand, HBV replication was significantly inhibited in the STAU1-knockdown stable line (Figures 3Q–3S). We further analyzed the effect of STAU1 on HBV cccDNA. As shown in Figure 3T, STAU1 overexpression significantly increased the transcriptional activity of cccDNA without altering its level. Furthermore, the knockdown of STAU1 significantly reduced the transcriptional activity of cccDNA while having no effect on its level. These results further suggest that STAU1 promoted HBV replication by enhancing cccDNA transcription.

### STAU1 binds to CP indirectly

**Figure 4:**
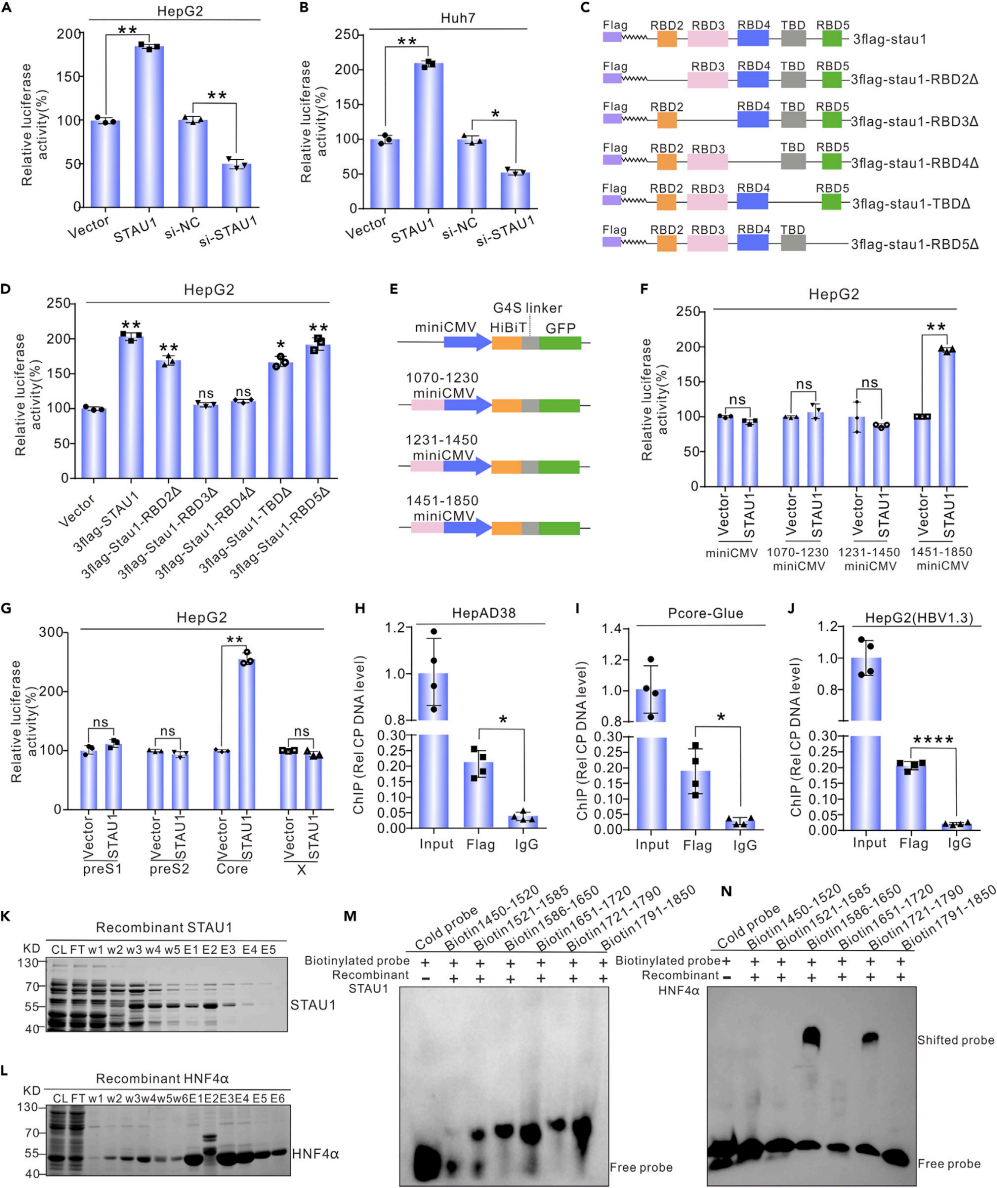
STAU1 enhances the activity of HBV core promoter (A and B) STAU1 increased the activity of HBV core promoter in HepG2 and HuH7 cells. pCH9-STAU1 or si-STAU1 was co-transfected with pCH9-PCore-Rluc into HepG2 cells and Huh7 cells, and renilla luciferase activity was detected after 48 h. (C and D) Characterization of the domains required for the effects of STAU1 on the HBV core promoter. Plasmids expressing different deleting mutants of STAU1 were constructed and co-transfected with pCH9-PCore-Rluc. Renilla luciferase activity was assayed after 48 h. (E) Schematic diagram of the constructs with the truncated HBV core promoter. Different core promoter regions were placed upstream of the mini-CMV promoter. The GFP gene fused with a HiBiT tag (Promega) was used as the reporter. (F) Identification of the regions in the core promoter required for the effects of STAU1. The indicated constructs and pSTAU1 or vector were co-transfected into HepG2 cells, and the luciferase signal was detected by the Nano-Glo HiBiT kit. (G) The effects of STAU1 on the activity of preS1, preS2, and X promoters. (H–J) ChIP assays for the interaction between STAU1 and core promoter. Plasmid 3flag-STAU1 was transfected into HepAD38, PCore-Gluc, and HBV1.3-transfected HepG2 cells, respectively. ChIP assays were conducted 2 days post-transfection. The data in (A to J) are represented as the mean ± SD from at least three independent experiments and analyzed with an unpaired *t*-test. ∗*p* < 0.05, ∗∗*p* < 0.01, ∗∗∗∗*p* < 0.0001, ns = non-significant. (K and L) Expression of recombinant STAU1 and HNF4α from *E. coli.* (M) and (N) EMSA assays to detect the interaction between STAU1/HNF4α and the HBV core promoter.

An increase in the level of pgRNA might indicate that the CP has been activated by STAU1. To test this hypothesis, an STAU1 construct or STAU1-targeting siRNA was co-transfected with a core-promoter reporter (pcore-Rluc). As expected (Figures 4A and 4B), STAU1 overexpression increased the luminescence signal, and STAU1 knockdown decreased it. We also evaluated whether STAU1 activates other HBV promoters (preS1, pre2, and X promoters) and found that STAU1 overexpression does not affect these promoters significantly (Figure 4G).

To identify domain(s) of STAU1 required for its effects on CP, we constructed five deleting mutants (Figure 4C). As shown in Figure 4D, the deletion of the RBD3 or RBD4 domain abrogated the effects of STAU1 on CP activity, indicating that these two domains are essential for its effects. To identify the regions in the HBV CP necessary for the interaction with STAU1, we created plasmids expressing different CP regions (Figure 4E) and performed luciferase assays. The findings revealed that STAU1 might enhance luciferase signal primarily via interacting with the CP region 1,451–1,850 (Figure 4F).

Activation of the CP by STAU1 suggested that it might directly or indirectly bind to the CP. Therefore, we tested this hypothesis with the ChIP assay. The p3flag-STAU1 plasmid was transfected into a stable cell line integrated with the HBV CP (HepG2-pCore-Gluc) and HepAD38 cells, respectively. Flag-antibody and IgG (control) were used to precipitate the disrupted genome, and CP DNA was detected by qPCR. The results showed that the HBV CP sequence was enriched by Flag-antibody compared with the IgG controls in both HepAD38 and HepG2-pCore-Glue cells (Figures 4H and 4I), indicating that STAU1 binds to the CP. The CP sequence is present in both the chromosome and episome of the two cell lines mentioned above. To see whether STAU1 binds to the episomal CP sequence, we co-transfected plasmid 3flag-STAU1 and HBV1.3 into HepG2 cells and performed ChIP experiments. The findings revealed that STAU1 and the episomal CP have an interaction (Figure 4J).

To test whether STAU1 binds to the CP directly, we performed electrophoretic mobility shift assays (EMSA) using recombinant STAU1 expressed from *E. coli* and seven biotin-labeled probes covering the CP sequence (HBV 1,450–1,850), with HNF4α as a positive control (Figures 4K–4L). However, electrophoretic shifted bands were not observed in the STAU1 group (Figure 4M) but in the HNF4α positive control group (Figure 4N), indicating that STAU1 does not bind to the HBV CP sequence directly.

### STAU1 binds to CP indirectly by interacting with TARDBP

**Figure 5:**
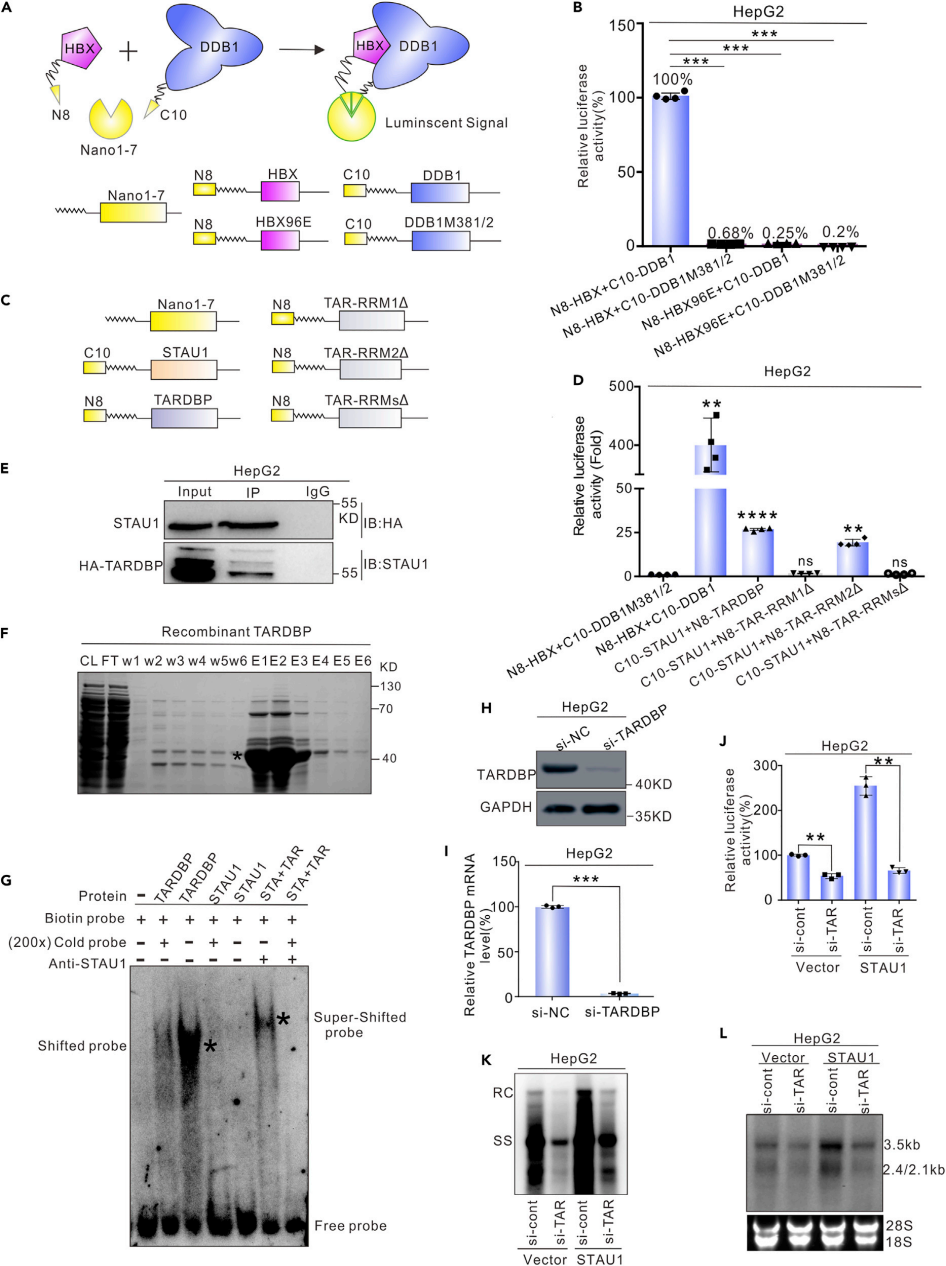
STAU1 indirectly binds to the core promoter via TARDBP (A) Operating principle of the tripartite NanoLuc system. (B) The tripartite Nanoluc system can be used to detect the interaction between HBx and DDB1. (C and D) The interactions between STAU1 and TARDBP or TARDBP mutants were assayed using the tripartite NanoLuc system. The data in (B) and (D) are represented as the mean ± SD of four independent experiments. Significance was tested with an unpaired *t* test. ∗∗*p* < 0.01, ∗∗∗*p* < 0.001, ∗∗∗∗*p* < 0.0001, ns = non-significant. (E) The interaction between STAU1 and TARDBP was assayed by co-IP. (F) Expression and purification of recombinant TARDBP protein from *E. coli.* (G) Detection of the interaction between STAU1, TARDBP, and core promoter by EMSA. (H and I) Knockdown efficiency of si-TARDBP was evaluated by qPCR and Western blot. (J) The si-Control (Lane1 and 3) or si-TARDBP (Lane2 and 4) were transfected into HepG2 cells. pCH9-STAU1 or the vector were co-transfected with pCore-Rluc into these cells after 48 h. After another 48 h, the activity of the core promoter was examined by renilla luciferase assays. The data in (I) and (J) are presented as the mean ± SD of three independent experiments and analyzed with an unpaired *t*-test. ∗∗*p* < 0.01, ∗∗∗*p* < 0.001. (K and L) pCH9-STAU1 or vector were co-transfected with HBV1.3 into TARDBP-depleted HepG2 cells and the intracellular HBV DNA and HBV RNA were detected by Southern and Northern blots.

Previous studies suggest that STAU1 interacts with TARDBP. An additional study also showed that TARDBP binds to the HBV CP (Makokha et al., 2019; Wang et al., 2008; Yu et al., 2012). These imply a possibility that binding of STAU1 to the CP might be mediated by TARDBP. To test this hypothesis, the interaction between STAU1 and TARDBP was analyzed by a protein–protein interaction (PPI) assay based on the tripartite Nanoluc luciferase (Ohmuro-Matsuyama and Ueda, 2018). This system was previously proven to be effective for detecting the interaction between two well-known proteins: HBx and DDB1 (Murphy et al., 2016). Methodologically (Figure 5A), C10 tag and N8 tag were fused to the N terminal of DDB1 and HBx, respectively. The two plasmids were co-transfected with a plasmid expressing Nano1-7 into HepG2 cells, and the luciferase activity was measured 48 h post-transfection. Compared with the controls (HBx and DDB1 mutants), wild type HBx and DDB1 complemented the tripartite Nanoluc efficiently, producing luminescence signals approximately 200-fold higher than the controls (Figure 5B). Next, this system was used to probe the interaction between STAU1 and TARDBP. Indeed, STAU1 and TARDBP complemented the tripartite Nanoluc effectively, leading to an approximate 23-fold increase in the luminescence signal compared with the controls (Figures 5C and 5D). Moreover, co-immunoprecipitation (co-IP) experiments showed that STAU1 was co-precipitated with HA-TARDBP by an anti-HA antibody (Figure 5E). Together, these results confirmed the interaction between STAU1 and TARDBP.

Next, we asked whether TARDBP could mediate the binding of STAU1 to the CP. To this end, we expressed and purified STAU1 and TARDBP recombinant proteins from *E. coli* (Figure 5F). EMSA experiments were conducted to analyze the interaction among STAU1, TARBDP, and HBV CP sequence. As shown in Figure 5G, TARDBP interacted with the HBV CP to form electrophoretic migration bands, whereas STAU1 did not bind to the HBV CP directly. When both STAU1 and TARDBP were present, electrophoretic super-migration bands were formed after the addition of the STAU1 antibody. Therefore, we concluded that TARDBP mediates the binding of STAU1 to the HBV CP.

To further explore the role of TARDBP in the effect of STAU1 on HBV replication, a TARDBP-targeting siRNA was designed and validated (Figures 5H and 5I). In the presence of the siRNA, the effects of STAU1 overexpression on both HBV CP activity and HBV replication were significantly reduced or even vanished (Figures 5J–5L), indicating that TARDBP plays an important role in the STAU1-mediated effect on HBV replication.

### STAU1 recruits the SAGA transcription coactivator complex

**Figure 6:**
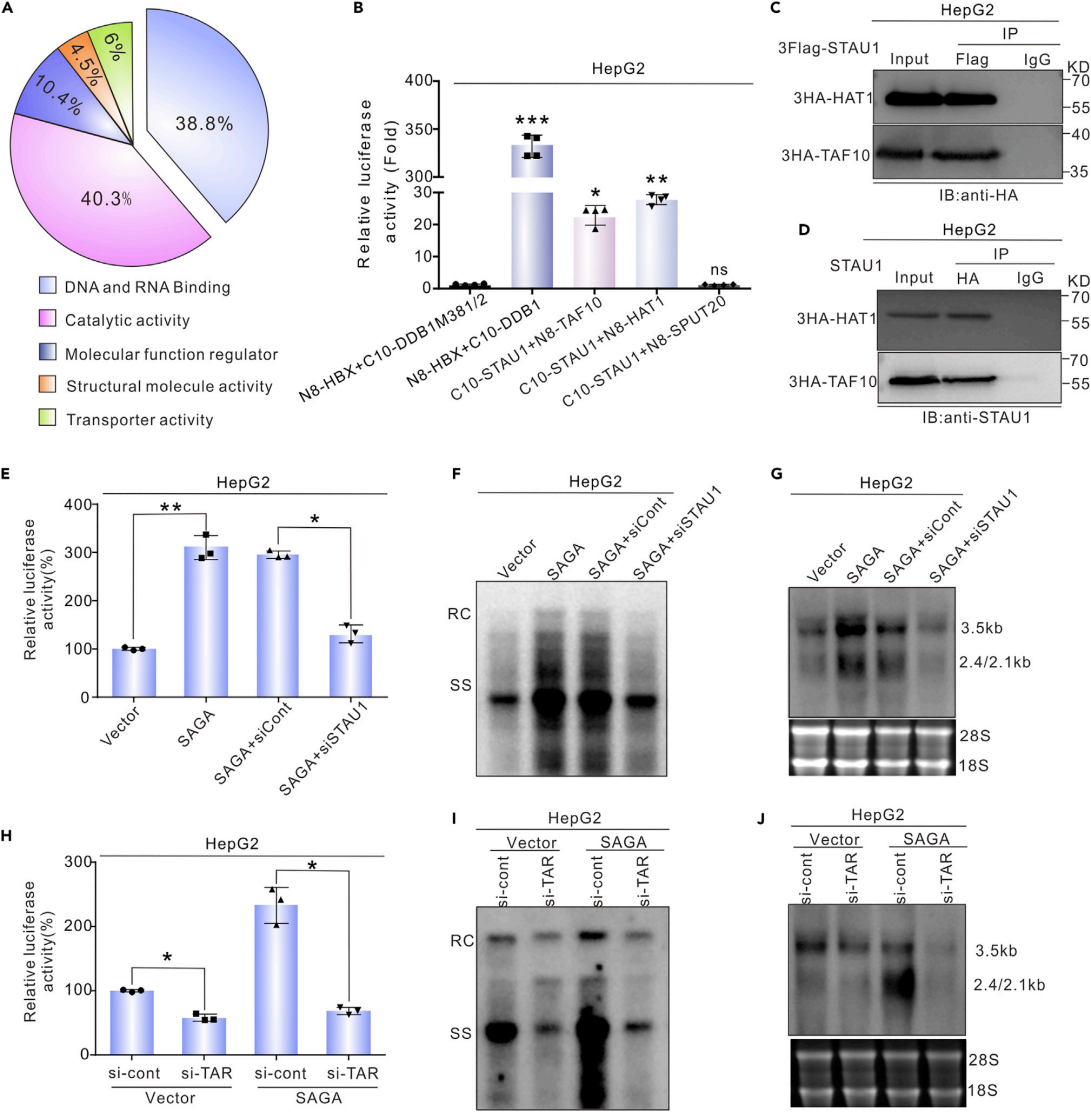
STAU1 recruits the SAGA transcription coactivator complex (A) Functional classification of the STAU1-interacting proteins identified by LC-MS. (B) Detection of the interactions between STAU1 and HAT1, TAF10, and SPUT20 by the tripartite Nanoluc system. The data are from four independent experiments and analyzed with an unpaired *t*-test. ∗*p* < 0.05, ∗∗*p* < 0.01, ∗∗∗*p* < 0.001, ns = non-significant. (C) The interactions between STAU1 and HAT1 were detected by co-IP. (D) The interaction between STAU1 and HAT1 was detected by reverse co-IP. (E) STAU1 knockdown abrogated the effect of SAGA complex on the activity of core promoter. The si-Control or si-STAU1 were transfected into HepG2 cells. The vector or SAGA complex (HAT1/TAF10/SPUT10) was co-transfected with pCore-Rluc into these cells after 48 h, and the activity of core promoter was detected by Renilla luciferase assays after another 48 h. (F and G) STAU1 knockdown abrogated the effects of SAGA complex on HBV replication. The vector or SAGA complex was co-transfected with HBV1.3 into STAU1-depleted HepG2 cells and the intracellular HBV DNA and HBV RNA were detected by Southern and Northern blots. (H) TARDBP knockdown reduced the effect of SAGA complex on the activity of the core promoter. The data in (E and H) are presented as the mean ± SD of three independent experiments and analyzed with an unpaired *t* test. ∗*p* < 0.05, ∗∗*p* < 0.01. (I and J) TARDBP knockdown reversed the effect of SAGA complex on HBV replication.

To analyze the mechanism by which STAU1 activates the transcription of the CP, we first searched for the interaction proteins of STAU1 by mass spectrometry. Plasmid 3flag-STAU1 was transfected into HepG2 cells, and the proteins immunoprecipitated by an anti-Flag antibody or an IgG control were then analyzed with mass spectrometry. Of the detected proteins, 404 were only identified in the anti-Flag IP samples (Figure 6A; Table S5). Among these proteins, HAT1, TAF10 and SPUT20 belong to the SAGA (Spt-Ada-Gcn5-acetyltransferase) transcription coactivator complex, which is required for all regulated transcription involving RNA polymerase II (Soffers et al., 2020; Wang et al., 2020). We speculated that STAU1 might recruit the SAGA transcription coactivator complex to the vicinity of the CP to activate HBV transcription. To test this hypothesis, the interaction between STAU1 and HAT1 or TAF10 or SPUT20 was assessed using the tripartite Nanoluc system. As shown in Figure 6B, N8-HAT1 and N8-TAF10 plus STAU1 produced luminescence signals that were more than 20 times greater than those from the control. However, N8-SPUT20 did not restore Nanoluc activity. The co-IP experiments further confirmed the interactions between STAU1 and HAT1, and STAU1 and TAF10 (Figures 6C and 6D). These results support the idea that STAU1 recruits the SAGA transcription coactivator complex to the CP.

Next, we analyzed the function of SAGA in the activation of HBV replication by STAU1. The overexpression of SAGA complex components (HAT1/TAF10/SPUT20) significantly increased HBV CP activity and HBV replication (Figures 6E–6G). However, when STAU1 was knocked down, the effects of the SAGA complex disappeared (Figures 6E–6G). The relationship between TARDBP and the SAGA complex was studied further. TARDBP knockdown reversed the effects of the SAGA complex on HBV replication (Figures 6H–6J), a phenomenon similar to that observed in STAU1 knockdown experiments. Taken together, these data suggest that the SAGA complex interacts with STAU1 to affect HBV replication.

### STAU1 interacts with HBx and enhances its stability

**Figure 7:**
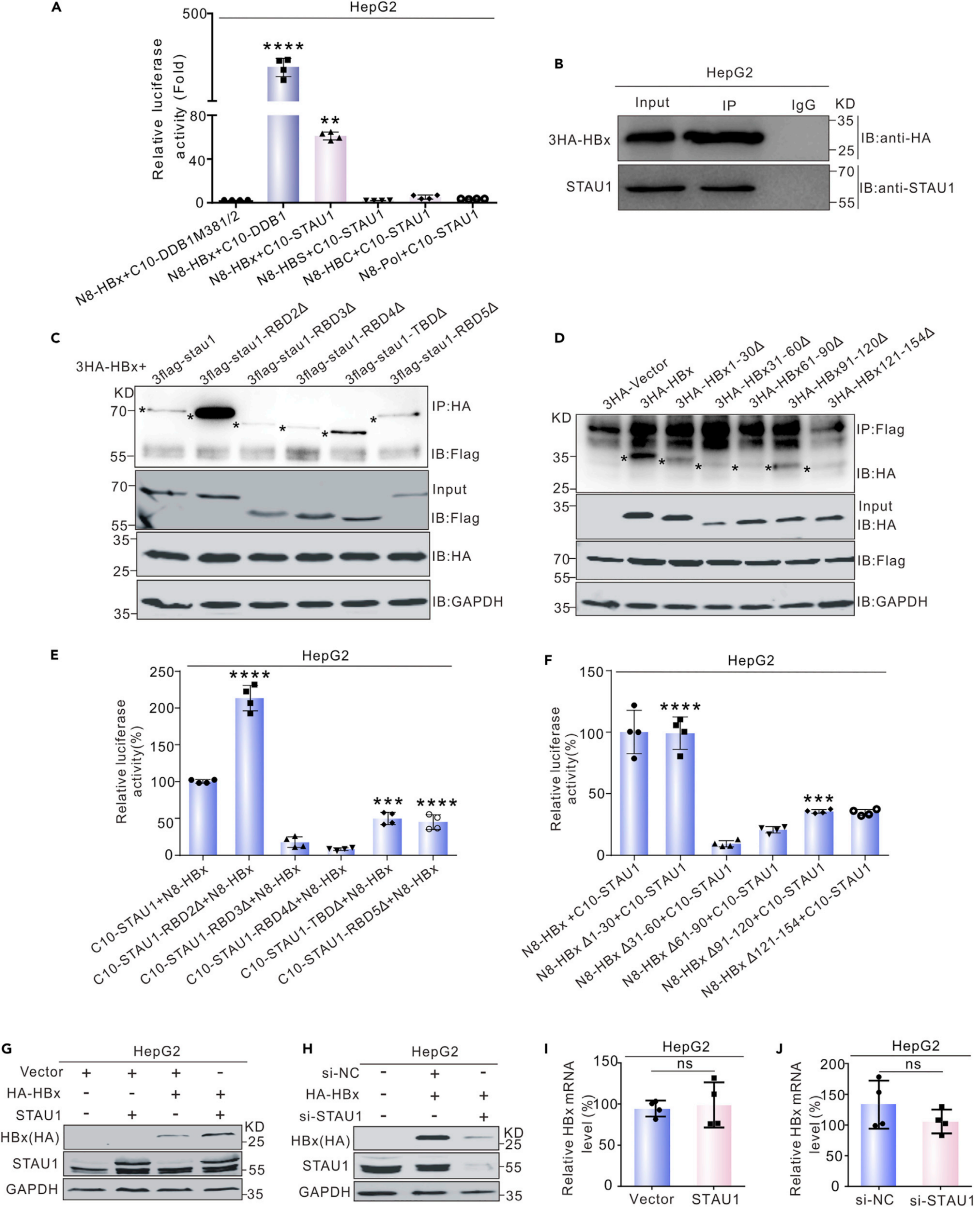
STAU1 interacts with HBx (A) Screening of HBV proteins interacting with STAU1 by the tripartite Nanoluc system. (B) Detection of the interaction between HBx and STAU1 by co-IP. (C) Analysis of the interactions between STAU1 mutants and HBx by co-IP. (D) Co-IP assays for the interactions between HBx mutants and STAU1. (E) Characterization of STAU1 domains required for its interaction with HBx by the tripartite Nanoluc system. (F) Characterization of HBx sequences required for its interaction with STAU1 by the tripartite Nanoluc system. The data in (E) and (F) are shown as the mean ± SD from four independent experiments. Significance was tested with an unpaired *t* test. ∗∗∗*p* < 0.001, ∗∗∗∗*p* < 0.0001. (G–J) The effects of STAU1 overexpression (G and I) and knockdown (H and J) on the level of HBx protein and mRNA were analyzed. The data in (I and J) are resented as the mean ± SD of three independent experiments.

As previously reported, STAU1 promoted the replication of a variety of viruses, such as HIV, HCV, and Ebola virus, by interacting with viral proteins (Chatel-Chaix et al., 2007; Dixit et al., 2016; Fang et al., 2018). We asked whether STAU1 interacts with viral proteins of HBV to promote HBV replication. To this end, we detected the interactions between STAU1 and four HBV proteins, including core, polymerase, S protein, and X protein, using the tripartite Nanoluc assay. Interestingly, only HBx among the four proteins interacted with STAU1, showing a 28-fold increase in the luminescence, compared with the controls (Figure 7A). Moreover, STAU1 co-precipitated with HBx in both the co-IP and reverse co-IP experiments (Figure 7B), confirming the interaction between STAU1 and HBx.

To identify domain(s) of STAU1 required for the interaction with HBx, we constructed five mutants and performed tripartite Nanoluc assays. As shown in Figure 7E, the deletion of the TBD or RBD5 domains partially reduced the luminescence signals. Removal of the RBD3 or RBD4 domain entirely disrupted the interaction. Surprisingly, the deletion of the STAU1 RBD2 domain significantly enhanced the luminescence signals by approximately two-fold compared with the wild type STAU1. In line with these results, IP experiments demonstrated that deletion of the RBD3 or RBD4 domain weakened the interaction (Figure 7C). Again, the deletion of the STAU1 RBD2 domain significantly increased the amount of HA-HBx co-precipitated. These results suggest that RBD3 and RBD4 are necessary for the interaction between STAU1 and HBx and RBD2 somehow inhibits the interaction.

To identify the regions in HBx required for the interaction with STAU1, we constructed a series of plasmids expressing HBx mutants and performed tripartite Nanoluc assays. As shown in Figure 7F, the deletion of HBx 1-30 aa (amino acid) had no effect on the interaction between STAU1 and HBx. The absence of HBx91-120aa moderately reduced the interaction. However, when HBx 31-60, 61-90, or 121-154 were removed, the luminescence signals were totally abrogated. Similar results were obtained from the IP experiments (Figure 7D). These findings indicate that HBx 31-60, 61-90, and 121-154 aa are essential for the interaction between HBx and STAU1.

Next, we asked what functions STAU1 might exert after binding to HBx. The levels of HBx were detected under conditions of STAU1 overexpression or knockdown. Interestingly, the overexpression of STAU1 increased the protein level of HBx, both in HepG2 (Figure 7G). In contrast, the knockdown of STAU1 reduced HBx protein level significantly (Figure 7H). The changes in HBx level could not be explained by the changes in HBx mRNA level (Figures 7I and 7J). Alternatively, it is possible that STAU1 regulated HBx levels by affecting its stability.

**Figure 8:**
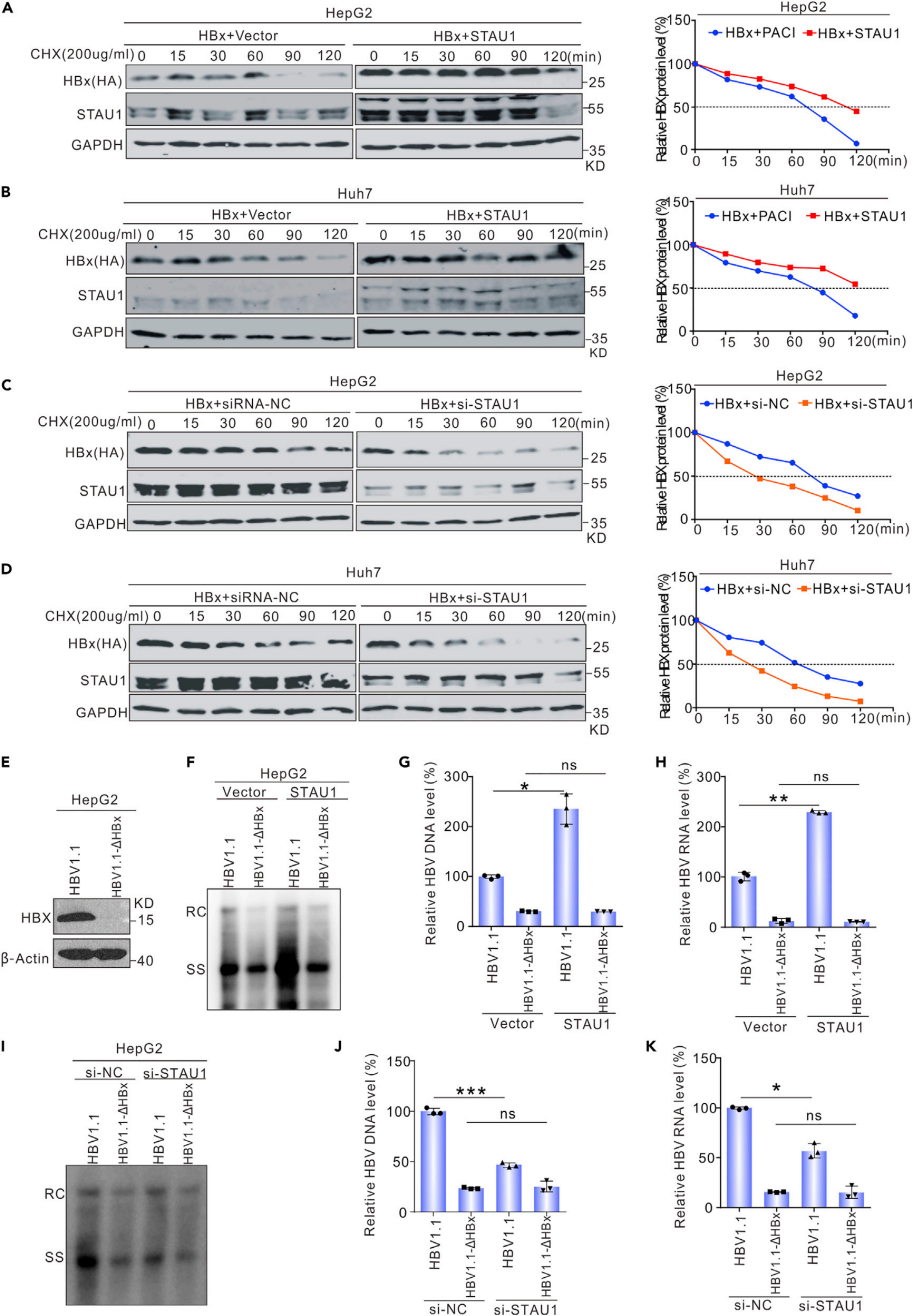
STAU1 enhances the level of HBx by stabilizing HBx (A–D) Influences of STAU1 on the stability of HBx. HepG2 (A) and Huh7 (B) were transfected with plasmid pCH9-STAU1 or vector. Cells were treated with cycloheximide (200 μg/mL of final concentration) after 48 h and lysed at different time points. The protein level of HBx was detected by Western blot. HepG2 (C) and Huh7 (D) were transfected with si-STAU1 or si-control. After being treated with cycloheximide, cells were lysed for Western blot. (E–K) (E) Plasmid HBV1.1 and HBV1.1-HBxΔ were transfected into HepG2 cells and HBx was detected by Western blot. pSTAU1 or vector was co-transfected with HBV1.1 or HBV1.1-HBxΔ into HepG2 cells. After 5 days, the level of intracellular HBV DNA was detected by Southen blot (F) and qPCR (G), and the level of HBV RNA was detected by RT-PCR (H) si-STAU1 or si-control was co-transfected with HBV1.1 or HBV1.1-HBxΔ into HepG2 cells. The level of HBV DNA was detected by Southen blot (I) and qPCR (J), and the level of HBV RNA was detected by RT-PCR (K). The data in (G), (H), (J), and (K) are shown as the mean ± SD from three independent experiments. Significance was tested with an unpaired *t* test. ∗*p* < 0.05, ∗∗*p* < 0.01, ∗∗∗*p* < 0.001, ns = non-significant.

To test this hypothesis, the half-life of the HBx protein was assessed through the cycloheximide chase assay. The p3HA-HBx and pSTAU1 plasmids or the control vector were co-transfected into HepG2 cells and Huh7 cells. After 48 h post-transfection, the cells were exposed to cycloheximide (200 μg/mL) for different periods, up to 3 h. Indeed, the overexpression of STAU1 extended the half-life of HBx (Figures 8A and 8B), whereas the knockdown of STAU1 decreased the half-life (Figures 8C and 8D). These results suggest that STAU1 enhances the stability of HBx.

To study the role of HBx in the effects of STAU1 on HBV replication, we co-transfected STAU1 and an HBV1.1 plasmid, in which the transcription of pgRNA is driven by the CMV-IE promoter (to rule out the effects of transcription), or an HBV1.1-ΔHBx plasmid, which does not express HBx gene owing to mutations introduced into its ORF (Figure 8E), into HepG2 cells. The results revealed that STAU1 overexpression enhanced HBV replication from HBV1.1 but not from HBV1.1-ΔHBx (Figures 8F–8H). In contrast, STAU1 knockdown only suppressed HBV replication from HBV 1.1 but not HBV1.1-ΔHBx (Figures 8I–8K). These findings indicate that HBx plays a role in the effects of STAU1 on HBV replication. We further examined the effects of STAU1 on Smc5 in the presence and absence of HBx, since HBx promotes HBV transcription by facilitating the degradation of restriction factors Smc5/6 (Decorsiere et al., 2016; Murphy et al., 2016). Consistent with previous reports, both HBx overexpression and HBV1.1 transfection lowered the levels of Smc5 (Figures S2A–S2D). Smc5 levels were further reduced by STAU1 overexpression (Figures S2A and S2C), and STAU1 knockdown restored them (Figures S2B and S2D). However, in the absence of HBx, the overexpression or knockdown of STAU1 had no effect on Smc5 levels (Figures S2E and S2F). These results confirm that STAU1 regulates Smc5 levels via HBx.

STAU1 inhibits the proteasome-mediated degradation of HBx.

**Figure 9:**
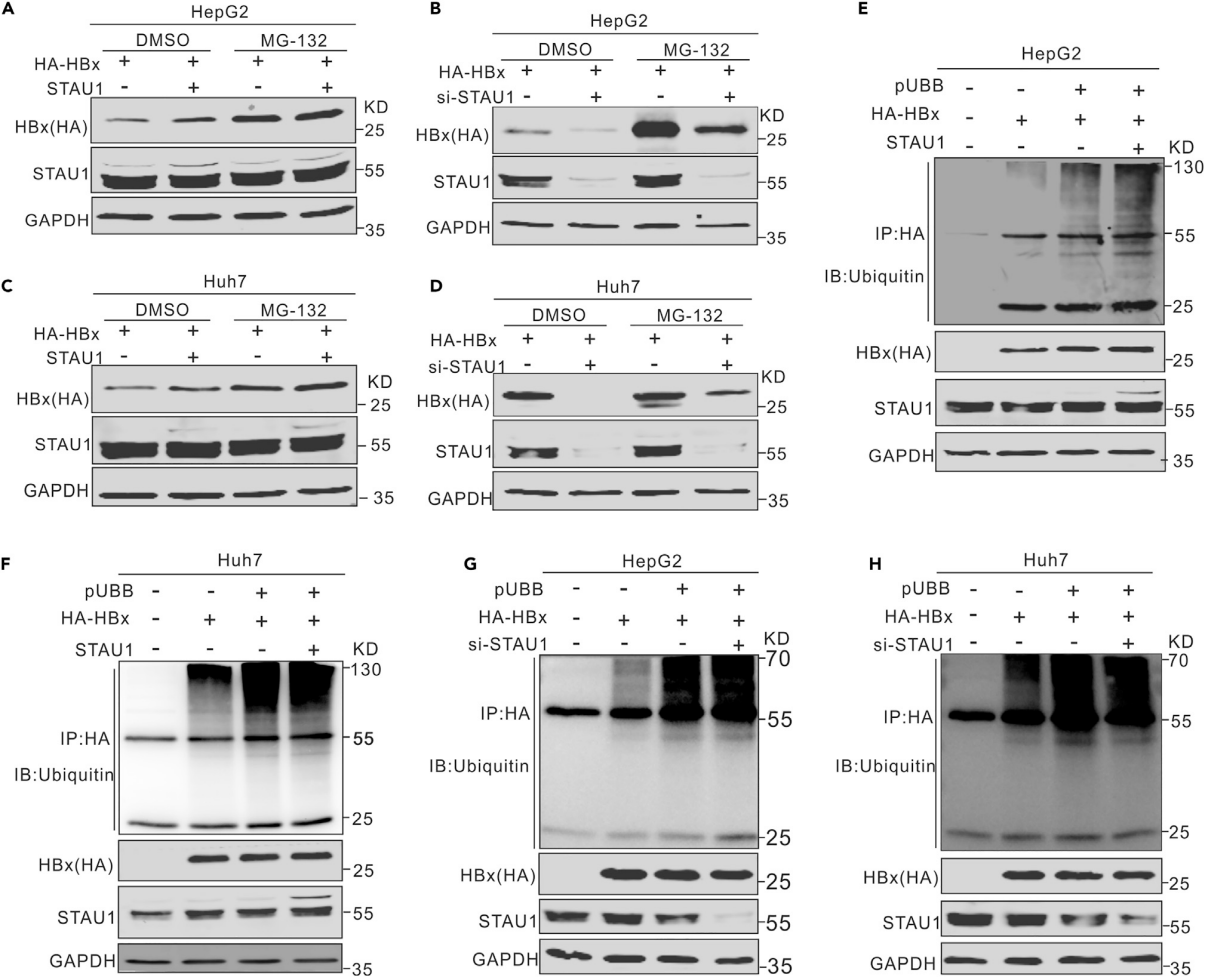
STAU1 inhibits the degradation of HBx in a ubiquitylation-independent manner (A–H) Plasmid pCH9-STAU1 and plasmid 3HA-HBx were co-transfected into HepG2 cells (A) and Huh7 cells (C). After 48 h, cells were treated with DMSO or MG132 (10 μM of final concentration) for 6 h, and then lysed for Western blot analysis. The si-STAU1 or si-control and plasmid 3HA-HBx were co-transfected into HepG2 cells (B) and Huh7 cells (D). After being treated with DMSO or MG132 for 6 h, cells were lysed for Western blot analysis. Plasmids pCH9-STAU1, pUBB and 3HA-HBx were co-transfected into HepG2 cells (E) and Huh7 cells (F). The si-STAU1, pUBB, and 3HA-HBx were co-transfected into HepG2 cells (G) and Huh7 cells (H). These cells were treated with MG132 for 6 h, and then lysed for IP 48 h post-infection.

It was previously reported that HBx can be degraded through the proteasome pathway. To test whether STAU1 stabilizes HBx by inhibiting proteasome-dependent degradation, we transfected the p3HA-HBx and pSTAU1 plasmids or siRNA-STAU1 into HepG2 cells and Huh7 cells. Thereafter, the cells were treated with MG132. The results showed that MG132 treatment attenuated the effect of STAU1 overexpression (Figures 9A and 9C) and abolished the effect of STAU1 knockdown on HBx (Figures 9B and 9D). Moreover, ubiquitylation assays were performed to determine whether STAU1 affects the ubiquitylation of HBx. As shown in Figures 9E–9H, neither the overexpression nor knockdown of STAU1 significantly changed the ubiquitylation level of HBx. This indicated that the ubiquitylation machinery was not involved in STAU1-mediated stabilization of HBx.

## Disscusion

In this study, we established a PL method based on TurboID to examine DNA-binding proteins. The method largely relies on recruiting promiscuous enzymes to the DNA sequence of interest. dCas9 can be guided to specific DNA sequence by sgRNA; thus, dCas9 fused with APEX2 has been successfully used in defining sub nuclear proteomic landscapes at genomic elements, and in discovering proteins associated with a predefined genomic locus (Gao et al., 2018; Myers et al., 2018). Herein, we provid an alternative strategy for recruiting promiscuous enzymes to specific DNA. A DNA sequence, known to bind with high affinity to an exogenous protein (EP), was placed next to the DNA of interest. A promiscuous enzyme, if fused to EP, then can be recruited to the DNA and labeling neighboring proteins, including those bound to the adjacent DNA. We provided a proof of concept of this strategy by placing TRE beside the HBV CP. It is noteworthy that another point of consideration for a successful PL is false positivity. Part of the false positivity arises from the proteins labeled by the enzymes that are not located at the targeting DNA sequence. To address the issue (false positivity), we separated TurboID from tTA, inspired by the work of Chojnowski et al. (2018), in which BioID was kept separate from the protein of interest. In the current study, we fused tTA with FRB and TurbolD with FKBP12. With this design, proteins labeled by FKBP12-TurboID in the absence of dimerization of FKBP12-TurboID and FRB-tTA would be subtracted as controls, thus reducing the rate of false positivity. The results revealed 42 candidate genes, which was a reasonable starting point for narrowing down to the real targets. Of note, among the 42 genes, 4 have been reported to be involved in the regulation of HBV, including S100A8 (Zhao et al., 2020), SRSF10 (Chabrolles et al., 2020), MYH9 (Lin et al., 2020), ZC3HAV1 (Mao et al., 2013), and 6 related to other viruses, including AGFG1(Yu et al., 2005), ERLIN1(Whitten-Bauer et al., 2019), DDX10 (Williams et al., 2015), ABCF1 (Lee et al., 2013), SRCAP (Ghosh et al., 2000), and G3BP1(Yang et al., 2019b). It is interesting to test whether these six genes play a role in the replication of HBV. In conclusion, the high positivity of the mapping results emphasizes the effectiveness of the strategy.

We identified STAU1 as an HBV transcription-related factor by a functional screening of the PL-mapped genes. STAU1 belongs to the family of double-stranded RNA (dsRNA)-binding proteins, involved in mRNA translation (Dugre-Brisson et al., 2005), transport and localization (Martel et al., 2006; Miki et al., 2005), differentiation of embryonic stem cells (Gautrey et al., 2008), and STAU1-mediated decay (SMD) (Kim et al., 2005; Park and Maquat, 2013). In addition, STAU1 has been reported to facilitate the replication of HIV-1 (Chatel-Chaix et al., 2007), Influenza A virus (de Lucas et al., 2010), HCV (Dixit et al., 2016), and Ebola virus (Fang et al., 2018). Our results uncovered new functions of STAU1 in the replication of HBV. Results from both overexpression and knockdown experiments in different cell models suggest that STAU1 is a positive-regulator of HBV replication. The luciferase assays revealed that STAU1 enhanced HBV replication by increasing the activity of the HBV CP. Mechanistically, STAU1 indirectly bound to the HBV CP (1,805–1,850) by interacting with TARDBP. This was further verified by our data from co-IP, EMSA, and tripartite Nanoluc assays. A previous study also demonstrated the direct binding of TARDBP to the CP (Makokha et al., 2019). We further demonstrated that TARDBP mediates STAU1 to bind to the HBV CP and promote HBV replication. Furthermore, IP-MS indicated that STAU1 might recruit the SAGA transcription coactivator complex. Results from co-IP and tripartite Nanoluc assays validated the interaction between STAU1 and HAT1, and STAU1 and TAF10. However, there was no interaction between STAU1 and SPUT20. Possibly, SPUT20, as a component of the SAGA transcription coactivator complex, might be covered by other components in the complex hence blocking the binding of STAU1. Previous studies have demonstrated that HAT1 can upregulate HBV replication by epigenetically modifying the cccDNA minichromosome (Wang et al., 2015; Yang et al., 2019a). Consistently, our results confirmed that the SAGA complex can activate HBV replication. This effect, on the other hand, can be reversed by STAU1 knockdown. Moreover, TARDBP knockdown also reduces the impact of the SAGA complex on HBV replication. These findings suggest that the SAGA complex interacts with STAU1 to promote HBV replication. The SAGA complex contains acetyltransferases that acetylate nucleosomes at gene promoters *in vitro* and *in vivo* (Bonnet et al., 2014; Huang et al., 2022). One possibility is that STAU1 recruits the SAGA complex to the HBV CP as an effector to modulate CP activity by acetylating histones. Alternatively, the SAGA complex recruited may influence HBV replication by modifying the function of STAU1 or TARDBP via acetylation. As a result, more research into the relationship between STAU1, SAGA and HBV is required.

Interestingly, the findings showed that STAU1 also interacts with the HBx protein. This is not surprising, given that STAU1 has been reported to interact with various viral proteins (Chatel-Chaix et al., 2007; de Lucas et al., 2010; Fang et al., 2018). In characterizing domains of STAU1 required for its interaction with HBx, we observed a significant increase in the amount of co-precipitated HBx and in the luminescence signal in the tripartite Nanoluc assay, when the RBD2 domain was deleted. It is well known that the RBD2 domain helps STAU1 stay in the cytoplasm. Reasonably, deleting of the RBD2 might have increased the nuclear import of STAU1. It is still unclear whether this relocation of STAU1 to the nuclei accounted for the increased interaction. Alternatively, the deletion of RBD2 might have conferred some conformational benefits on the binding of HBx.

HBx is a 154 aa protein with rapid renewal and a short half-life (Schek et al., 1991). HBx can be degraded through the proteasome pathway in both typical ubiquitin-dependent (Hu et al., 1999) and ubiquitin-independent (Kim et al., 2008; Orlowski and Wilk, 2003) manners. Host factors can accelerate HBx degradation by stimulating the proteasome system (Kido et al., 2011), or enhance HBx stability through different mechanisms (Pang et al., 2007). The results herein clearly show that STAU1 functions as an HBx stabilizer by binding to HBx (Figures 9A–9D). Further studies suggest that STAU1 protects HBx from proteasome-mediated degradation in a ubiquitin-independent manner. We speculated that STAU1 may interfere with the interactions between HBx and 20S proteasome subunits such as PSMA1, PSMA3, and PSMA7 (Minor and Slagle, 2014). However, we did not observe such an effect of STAU1 on these interactions in IP assays (data not shown). Therefore, the detailed mechanism of STAU1-mediated HBx stabilization needs to be explored further. Furthermore, we found that STAU1 functions partially via HBx because STAU1 promotes HBV1.1 (driven by the CMV-IE promoter rather than the HBV CP) replication but fails to affect HBV replication from HBV1.1 that does not express HBx. It is reasonable that stabilized HBx would facilitate the degradation of restriction factors Smc5/6 (Decorsiere et al., 2016; Murphy et al., 2016), and hence increase cccDNA transcription. Indeed, in the presence of HBx, STAU1 overexpression reduced Smc5 levels more than HBx overexpression or HBV1.1 transcription alone (Figure S2). This effect was lost in the absence of HBx, indicating that STAU1 affects Smc5 via HBx.

In summary, our study developed a TurboID-based PL method for identifying transcription regulatory factors. Using this technique, STAU1 was discovered to be a positive regulator of HBV transcription. Mechanistically, STAU1 promotes HBV transcription and replication in two different ways. First, STAU1 indirectly binds to the CP via TARDBP and recruits the SAGA transcription coactivator complex to upregulate CP activity. Second, STAU1 boosted HBV transcription by increasing the stability of HBx. These findings deepen our understanding of how HBV transcription is regulated by host factors.

### Limitations of the study

Whereas it provides evidence that STAU1 may serve as a potential anti-HBV target by positively regulating HBV replication, the current study has a few limitations. First, our results indicate that STAU1 can interact with the SAGA complex to regulate HBV replication. However, the available evidence is insufficient to determine whether SAGA is a downstream effector of STAU1 or vice versa. More research is needed to fully comprehend how STAU1, SAGA, and HBV interact. Second, the physiological role of STAU1 in HBV replication regulation has yet to be determined *in vivo*. Experiments in animal models are needed to further test the effects of STAU1 on HBV replication. Finally, in order to apply our findings, inhibitors targeting STAU1 can be screened and evaluated. The discovery of STAU1 inhibitors that limit HBV replication will strengthen the argument that STAU1 plays a key role in HBV replication.

## STAR★Methods

### Key resources table

**Table:** 

REAGENT or RESOURCE	SOURCE	IDENTIFIER
**Antibodies**		

Flag Tag antibody mouse	Sigma-Aldrich	Cat#P2983; RRID: AB_439685
HA Tag Monoclonal Antibody	Thermo Fisher Scientific	Cat#26183; RRID: AB_2533056
STAU1 antibody	Abclonal	Cat#A4131; RRID: AB_2765518
TARDBP antibody	Santa Cruz Biotechnology	Cat#sc-102127; RRID: AB_2200515
HNF4A Rabbit Polyclonal Antibody	Sigma-Aldrich	Cat#AV31946; RRID: AB_1850814
Hepatitis B Virus X antigen antibody	Abcam	Cat#ab39716; RRID: AB_880382
Anti-Ubiquitin Antibody	Santa Cruz Biotechnology	Cat#sc-8017; RRID: AB_628423
GAPDH Mouse Monoclonal Antibody	Beyotime	Cat#AG019; RRID: AB_2861160
β-Actin Mouse Monoclonal Antibody	Beyotime	Cat#AF0003; RRID: AB_2893353)
Streptavidin-HRP antibody	Cell Signaling Technology	Cat#3999; RRID: AB_10830897)
SMC5 antibody	GeneTex	Cat#GTX115669; RRID:AB_10632730
IRDye® 800CW Goat anti-Rabbit IgG (H + L)	Licor Biosciences	Cat#C50331-05
IRDye® 800CW Goat anti-Mouse IgG (H + L)	Licor Biosciences	Cat#C50113-06
light/heavy chain specific secondary antibodies	Jackson	Cat#211-032-171

**Bacterial and virus strains**		

DH5α Chemically Competent Cell	TSINGKE	Cat#TSC-C01
BL21 Chemically Competent Cell	TSINGKE	Cat#TSC-E06

**Chemicals, peptides, and recombinant proteins**		

DTT	Thermo Scientific	Cat#D1532
ATP	New England Biolabs	Cat#P0756S
Tango Buffer (10×)	Thermo Scientific	Cat#BY5
BsmbI-V2	New England Biolabs	Cat#R0739S
T7 DNA ligase	New England Biolabs	Cat#M0318S
T5 exonuclease	New England Biolabs	Cat#M0363
PrimeSTAR Max DNA Polymerase	TAKARA	Cat#R045B
IPTG	Beyotime	Cat#ST098
Pronase	Roche	Cat#10165921001
Micrococcal nuclease	New England Biolabs	Cat#M0247S
MG132	MCE	Cat#HY-13259
Blasticidin	Sigma-Aldrich	Cat#SBR00022
STAU1 recombinant protein	In-house	N/A
TARDBP recombinant protein	In-house	N/A
HNF4α recombinant protein	In-house	N/A

**Critical commercial assays**		

Lipofectamine 3000 Transfection Reagent	Thermo Scientific	Cat#L3000015
TRIzol Reagent	Thermo Scientific	Cat#15596018
protein A/G Mix Magnetic Beads	Thermo Scientific	Cat#88803
Streptavidin-coated magnetic beads	Thermo Scientific	Cat#88817
Renilla luciferase assay system	Promega	Cat#E2810
Nano-Glo HiBiT Lytic Detection Reagent	Promega	Cat#N3030
Nano-Glo luciferase assay system	Promega	Cat#N1110
His-tag Protein Purification kit	Beyotime	Cat#P2229S
DIG DNA labeling and detection kit	Roche	Cat#11745832910
DIG Northern starter kit	Roche	Cat#12039672910
LightShift Chemiluminescent EMSA Kit	Thermo Scientific	Cat#20148
QIAamp DNA Mini Kit	QIAGEN	Cat#51304

**Deposited data**		

TurboID MS data (Table S4)	This paper	N/A
STAU1-interacting proteins MS data (Table S5)	This paper	N/A

**Experimental models: Cell lines**		

HEK293FT	ATCC	N/A
HEK293	ATCC	N/A
HepG2	ATCC	N/A
Huh7	ATCC	N/A
HepAD38	In-house	N/A
HepG2-NTCP	In-house	N/A

**Oligonucleotides**		

All primer sequences are listed in Table S1	TSINGKE	N/A
All siRNA sequences are listed in Table S2	TSINGKE	N/A
All probe sequences are listed in Table S3	TSINGKE	N/A

**Recombinant DNA**		

Plasmid pCH9/3091 (HBV1.1)	In-house	N/A
Plasmid HBV1.1-HBxΔ	In-house	N/A
Plasmid HBV1.3	In-house	N/A
Plasmid lenti-Cas9-BLAST	Addgene	Cat#125592
Plasmid LKO.1-EGFP-Blast	In-house	N/A

**Software and algorithms**		

GraphPad Prism 5	GraphPad Software	https://www.graphpad.com/
DNAMAN8	N/A	N/A
CorelDRAW 2017	N/A	N/A
Primer Premier 6	N/A	N/A

### Resource availability

#### Lead contact

Further information and requests for resources and reagents should be directed to and will be fulfilled by the lead contact, Jie-li Hu (102564@cqmu.edu.cn).

#### Materials availability

The plasmids are available upon request. This study did not generate other unique reagents.

### Experimental model and subject details

#### Cell lines and cell culture

HepG2, Huh7, HEK293 and HEK293FT were purchased from ATCC. HepG2-NTCP cell line was kindly provided by Professor Ningshao Xia (Xiamen University, China). HepAD38 was established by Robert lab and kindly gifted by Dr. Hong Tang (Sichuan University, China). All cells were maintained in Dulbecco’s modified Eagle’s medium supplemented with 10% (v/v) fetal bovine serum at 37 °C under 5% CO_2_.

### Method details

#### Plasmids construction

All the plasmids except the lentiviral plasmids were constructed through the Golden Gate cloning method (Engler et al., 2008, 2009), based on the backbone of pCH9/3091 (Nassal, 1992). Notably, the pTRE-PCORE plasmid had a sequence from the Tetracycline Response Element (TRE) and the HBV core promoter/enhancer (1450-1850). The p3Flag-tTA-FRB plasmid was constructed by fusing fragments of 3xFlag, the tetracycline transactivator protein (tTA) and fragments of FKBP-rapamycin binding (FRB). In addition, plasmid FKBP12-TurboID expressed the rapamycin-binding protein (FKBP12) fused with TurboID. The HBV1.3 plasmid, containing a 1.3-fold HBV genome was also a kind gift from Dr. Xuesen Zhao. The HBV1.1-HBXΔ was constructed by introducing a stop codon at the beginning of the HBx CDS based on the plasmid pCH9/3091 (HBV1.1) (Cheng et al., 2021). Moreover, the pcore-Rluc plasmid expressed renilla luciferase under the control of the HBV core promoter. Furthermore, plasmids 3Flag-STAU1, 3Flag-STAU1 mutants, 3HA-TARDBP, 3HA-TARDBP mutants, 3HA-HBx and 3HA-HBx mutants were constructed by fusing 3xFlag tag or 3xHA tag to the N terminus of corresponding genes, separated by G_4_S linkers with 47 amino acids in length. Finally, plasmids expressing C10-STAU1, C10-STAU1 mutants, C10-PSMA1, C10-PSMA3, C10-PSMA7, C10-PSMC1, C10-PSMC2, C10-PSMC3, N8-TARDBP, N8-TARDBP mutants, N8-Pol, N8-HBc, N8-HBs, N8-HBx and N8-HBx mutants were constructed by fusing the C10 tag or N8 tag to the N terminus of the genes, separated by G_4_S linkers. The plasmid miniCMV expresses a HiBit-GFP fusion protein driven by a miniCMV promoter. Plasmids 1070-1230miniCMV, 1231-1450miniCMV and 1451-1850miniCMV contain sequences from the HBV core promoter (indicated by the numbers) upstream of the miniCMV promoter. The Lentiviral plasmids (lenti-STAU1-Blast, lenti-Vector-Blast) were constructed based on the lenti-Cas9-BLAST vector. Plasmid pLKO.1-shSTAU1-Blast and pLKO.1-shControl-Blast was constructed through the Golden Gate cloning method, based on the backbone of pLKO.1-EGFP-Blast. Primers are listed in Table S1.

#### Transfection of plasmids and siRNA

All the plasmids and siRNA were transfected using Lipofectamine 3000 Transfection Reagent (Thermo Scientific), according to the manufacturer’s instructions. The STAU1 siRNA and TARDBP siRNA were modified with cholesterol to improve the transfection efficiency.

#### Stable transduction with lentiviruses

Plasmid lenti-STAU1-Blast or lenti-shSTAU1-blast (7.5 μg) or the controls, plasmid PSPX2 (5.6 μg) and plasmid PMD2.0G (1.88 μg) were co-transfected into 293FT cells (in 10-cm dishes) using Lipofectamine3000 Transfection kit (Thermo Scientific). The media were harvested at 48 and 72 h after transfection. The media was concentrated by PEG-itTM Virus Precipitation Solution (5x) (SBI) and resuspended with PBS. HepG2-NTCP cells were infected by adding 300 μL lentiviral suspension. Cells were exposed to 10 μg/mL blasticidin (Sigma-Aldrich) 48 h after transduction. After a 7-day selection, the surviving cells were pooled and expanded for the experiments.

#### HBV preparation and infection

HBV particles were precipitated from HepAD38 culture medium using 6% PEG8000 then resolved in Opti-MEM at a 100-fold concentration. The viral titer was determined by measuring HBV DNA with qPCR assays. In addition, the HepG2-NTCP cells were seeded in collagen-coated plates and pretreated with 2.5% DMSO for 24 h, prior to HBV infection. Thereafter, the cells were inoculated with HBV (1000 genome equivalents per cell) in 4% PEG-8000 and 2.5% DMSO for 18 h. After 18 h inoculation, the cells were rinsed with PBS and replenished with DMEM complete medium containing 2.5% DMSO. The medium was changed every other day.

#### Biotin labeling with TurboID and MS analysis

For biotin labeling, transfected cells were treated with 500 μM biotin at 37°C for 1 h and 200 nM rapamycin at 37 °C for 12 h. The reaction was terminated by transferring the cells to ice and washing with ice-cold PBS. The cell pellets were collected and lysed using the RIPA lysis buffer. Streptavidin-coated magnetic beads (Thermo Scientific) were added to capture biotinylated proteins overnight at 4 °C. After washing, biotinylated proteins were eluted by boiling the beads with the protein loading buffer containing 20 mM DTT and 2 mM biotin. Finally, the proteins were analyzed through LC-MS/MS.

#### The luciferase activity assay

The activity of renilla luciferase was detected through the renilla luciferase assay system (Promega), following the protocol. HiBiT tag was detected by the Nano-Glo HiBiT Lytic Detection Reagent (Promega). The activity of Nanoluc luciferase was assessed by the Nano-Glo luciferase assay system (Promega) according to the manufacturers’ instructions. Moreover, the GloMax-Multi Jr detection system (Promega) was used to detect bioluminescence.

#### Expression and purification of recombinant STAU1, HNF4α and TARDBP

For recombinant protein expression, PQE30-STAU1, PQE30-HNF4α and PQE30-TARDBP were transformed into BL21 competent cells. The selected clones were inoculated in a culture medium (containing Ampicillin and Kanamycin) and cultured at 37 °C to achieve an OD600 of 0.7. IPTG was added to the medium (final concentration 1 mM) which was then cultured at 26 °C for 18 h, to induce protein expression. The medium was centrifuged at 4000 g for 20 min and then the bacterial pellet was collected. Finally, the recombinant proteins were purified using His-tag Protein Purification (Denaturant-resistant) Kit (Beyotime), according to the manufacturer’s instructions.

#### HBV DNA, RNA and cccDNA assay

For HBV core DNA extraction, cells in 12-well plates were lysed 5 days after transfection with lysis buffer (10 mM Tris-Hcl (pH 8.0), 0.2% NP-40 and 1 mM EDTA). The cell debris was removed by centrifugation. Thereafter, 5 μL of 200 mM CaCl_2_ and 1 μL micrococcal nuclease (NEB) were added into the supernatant for 1 h at 37 °C. This reaction was stopped by adding 4 μL 0.5 M EDTA. Afterwards, 10 μL of 10 mg/mL pronase (Roche) and 10 μL of 10% SDS were added to the mixture, for 1 h at 37 °C. The mixture was then extracted with phenol, precipitated with ethanol, and dissolved with water. In addition, the samples were electrophoresed using 1.2% agarose gel then transferred onto Nylon membranes (Roche). The membranes were hybridized and then detected using a DIG DNA labeling and detection kit (Roche), according to the manufacturer’s instructions.

For HBV RNA assay, cells in 12-well plates were lysed using the TRIzol Reagent (Thermo Scientific) to extract total RNA, according to the manufacturer’s instructions. Additionally, the RNA samples were resolved using 1.2% gel containing 2% formaldehyde, after which they were transferred onto Nylon membrane (Roche). The HBV RNAs on the membranes were detected using a DIG Northern starter kit (Roche), following the manufacturer’s protocol.

cccDNA was detected using a T5 exonuclease hydrolysis-based method as described previously (Qu et al., 2018). Briefly, HepG2-NTCP cells were cultured in a 6-cm dish for 7 days after infection with HBV. The total DNA were extracted using the genomic DNA extraction kit (QIAGEN). The DNA samples were digested in the 10 μL reaction system which containing 5 μL genomic DNA, 3.5 μL ddH_2_O, 1 μL 10× reaction buffer, 0.5 μL T5 exonuclease (NEB), and then incubated at 37 °C for 1 h and 70 °C for 20 min. Digested samples were subjected to cccDNA qPCR. The levels of β-Globin in the undigested samples were used to normalize the inputs from different samples.

#### qPCR and RT-PCR analysis

The HBV core DNA was quantified using the SYBR Green qPCR Super Mix (Thermo Scientific), following the manufacturer’s protocol. On the other hand, the pgRNA assay was conducted by reverse transcribing total RNA to cDNA using the FastKing RT Kit (TIANGEN), after which the cDNA was quantified. The primer sequences were shown in Table S2.

#### Western blotting and the Co-immunoprecipitation (Co-IP) assay

In order to conduct the Western blot assay, the cells were lysed using the RIPA buffer (Beyotime) and protein concentration was determined using the BCA Protein Assay kit (Beyotime). In addition, the protein samples were analyzed through SDS-PAGE gels and then transferred onto PVDF membranes (Roche). The membranes were probed using the primary antibodies. Finally, the membranes were visualized by IRDye-conjugated secondary antibodies (Licor Biosciences) using an Odyssey CLx system (Licor Biosciences).

For biotinylated protein detection, the protein samples were transferred onto PVDF membranes. After blocking, the membranes were directly incubated with Streptavidin-HRP antibody (Solarbio) for 1 h at room temperature. The membranes were visualized using the ECL Western Blotting Substrate (Solarbio) and a Fusion FX5 system (Vilber Lourmat).

For co-IP assay, the protein extracts were immunoprecipitated with appropriate primary antibodies or IgG at 4°C overnight. This was followed by incubating the samples for 2 h at 37 °C, in the presence of protein A/G Mix Magnetic Beads (Thermo Scientific). The beads were then washed 4 times and the immune complex was eluted with the sample loading buffer. The samples were subjected to SDS-PAGE and then analyzed by western blotting with mouse/rabbit anti-IgG or light/heavy chain specific secondary antibodies (Jackson 211-032-171).

#### The Electrophoretic mobility shift assay (EMSA)

The Electrophoretic mobility shift assay (EMSA) was performed using the LightShift Chemiluminescent EMSA Kit (Thermo Scientific) as per the manufacturer’s protocol. Briefly, four ul recombinant proteins, 20 fmol biotinylated probe or 4 pmol competitive probe were used in the EMSAs, unless stated otherwise. Additionally, the DNA-protein complexes were incubated at 37 °C for 30 min, then separated by electrophoresis in 5% gels with TBE buffer. The samples were transferred onto nylon membrane and detected by the kit according to the manufacturers’ instructions. The EMSA probe sequences are listed in Table S3.

### The chromatin immunoprecipitation (ChIP) assay

The Chromatin immunoprecipitation (ChIP) assay was performed as described previously (Spencer et al., 2003). Briefly, the 3Flag-STAU1 plasmid was transfected into cells, which were collected and cross-linked with 37% formaldehyde. The fixed cells were incubated with glycine to stop the formaldehyde reaction, after which they were lysed using the sucrose lysis buffer. Following this, the chromatin fragments were obtained by sonicating the cell lysates on ice. Moreover, the immunoprecipitated complexes were enriched with protein A/G magnetic beads in the presence of anti-Flag or IgG antibodies. The DNA-protein complexes were washed and then incubated with 5 M NaCl to relieve the cross-links. After digestion with proteinase K and RNase A, the DNA was purified and subjected to the qPCR assay. The region corresponding to the HBV core promoter was amplified using the ChIP primers (Table S1).

#### Immunofluorescence analysis (IFA)

Cells grown on coverslips in 24-well plates were fixed with 4% paraformaldehyde for 20 min, at room temperature (RT). Thereafter, they were permeabilized with 0.5% Triton for 30 min and blocked with 2.5% BSA blocking buffer for 1 h at RT. After being incubated with appropriate antibodies, the cells were stained with DAPI, and imaged under a confocal laser-scanning microscope (Leica).

#### Determination of HBx half-life and ubiquitylation assay

For HBx half-life assay, plasmid 3HA-HBx and plasmid STAU1 or siRNA-STAU1 were co-transfected into HepG2 cells. The vector plasmid or siRNA-NC was used as a negative control. After treatment with cycloheximide (100 μg/mL), the cells were collected at the indicated time points and lysed with RIPA buffer for western blot analysis.

The ubiquitylation assay was conducted by co-transfecting the UBB, 3HA-HBx and STAU1 plasmids or siRNA-STAU1 into HepG2 cells grown in 6-well plates. The vector plasmid or siRNA-NC was used as a negative control. The cells were incubated with 10 μM MG132 (MCE) for 6 h and then lysed with the RIPA buffer. The lysates were then incubated overnight with protein A/G Mix Magnetic Beads (Thermo Scientific) in the presence of the HA tag antibody, at 4 °C. Finally, the beads were washed 4 times and the eluted proteins were separated by SDS-PAGE and then detected via western blotting.

#### Quantification and statistical analysis

Each experiment was repeated at least three times and the data were presented as the Mean ± standard deviations (SD). Statistical analyses were performed using a two-tailed Student’s T test, using GraphPad Prism version 6 (GraphPad Software Inc.). p values <0.05 were considered to be statistically significant. Asterisks were used to indicate distinct p values: ∗, p < 0.05, ∗∗, p < 0.01, ∗∗∗, p < 0.001 and ∗∗∗∗, p < 0.0001.

## Data Availability

All data supporting the findings of this study are available within the paper and its supplemental information files. This paper does not report original code. Any additional information required to reanalyze the data reported in this paper is available from the lead contact upon request.

## Supplemental information

Document S1. Figures S1 and S2

Table S1. The primer sequences for this study, related to STAR Methods

Table S2. The siRNA sequences for this study, related to STAR Methods

Table S3. The EMSA probe sequences for this study, Related to STAR Methods

Table S4. Mass Spectrometry results of identifying host factors bound to HBV core promoter based on the TurboID method, related to Figure 2

Table S5. Mass Spectrometry results of the STAU1-interacting proteins identified by pull down assay, related to Figure 6
