# Supplementary material for: Identification of STAU1 as a regulator of HBV replication by TurboID-based proximity labeling
Source: iScience. 2022 May 18;25(6):104416. doi: 10.1016/j.isci.2022.104416 (PMC9156947; doi:10.1016/j.isci.2022.104416)
Supplement: Document S1. Figures S1 and S2 [file mmc1.pdf]

iScience, Volume *25*

Supplemental information

Identification of STAU1 as a regulator of HBV replication by TurboID-based proximity labeling

Xia-Fei Wei, Shu-Ying Fan, Yu-Wei Wang, Shan Li, Shao-Yuan Long, Chun-Yang Gan, Jie Li, Yu-Xue Sun, Lin Guo, Pei-Yun Wang, Xue Yang, Jin-Lan Wang, Jing Cui, Wen-Lu Zhang, Ai-Long Huang, and Jie-Li Hu

Supplementary Figures and Legends

Figure.S1

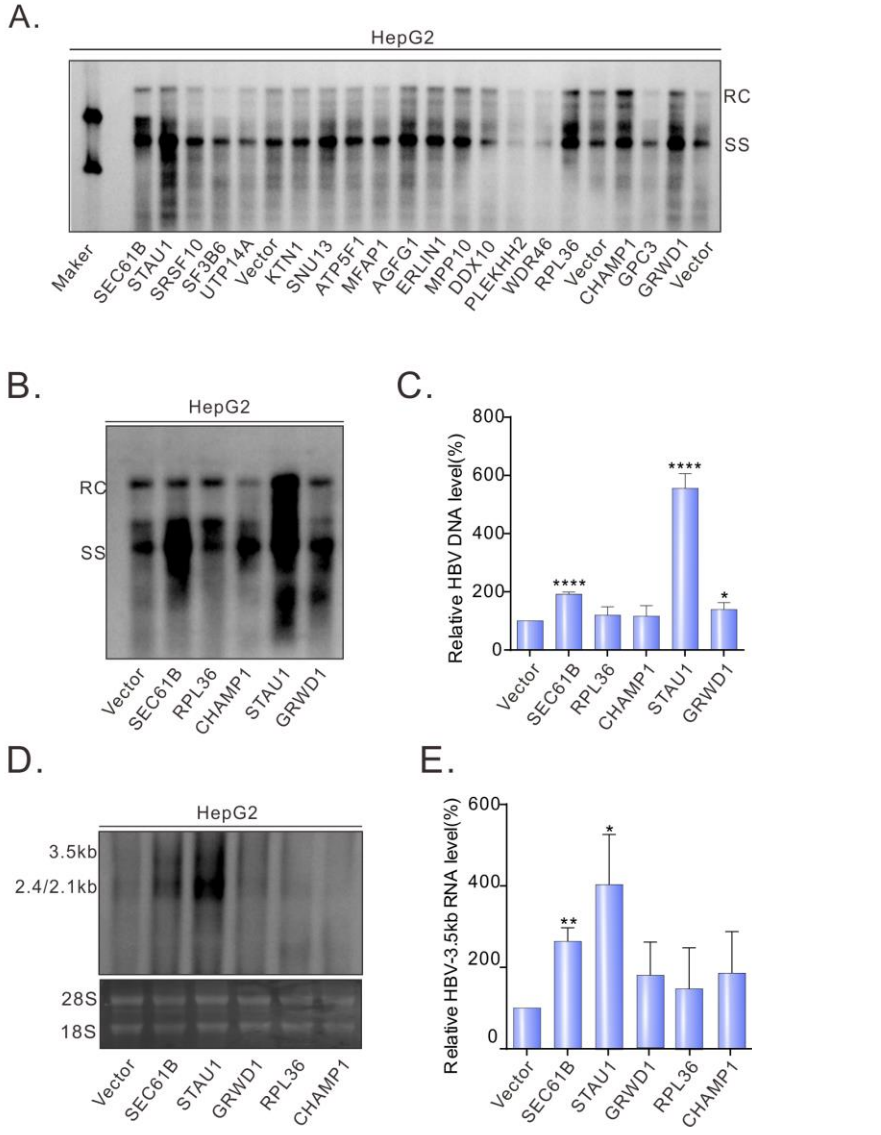

Figure S1. Second-round screening of the candidate genes, Related to Figure 2. (A) The effects of the 19 candidate genes on HBV replication. Expression plasmids of the candidate genes and plasmid pHBV1.3 were co-transfected into HepG2 cells and intracellular HBV DNA was detected by Southern blot. (B) to (E) The 5 genes selected (SEC61B, STAU1, RPL36, CHAMP1, GRWD1) were co-transfected with HBV1.3 into HepG2 cells. HBV DNA and RNA were extracted 5 days later and analyzed by Southern blot, Northern blot and qPCR.

Figure.S2

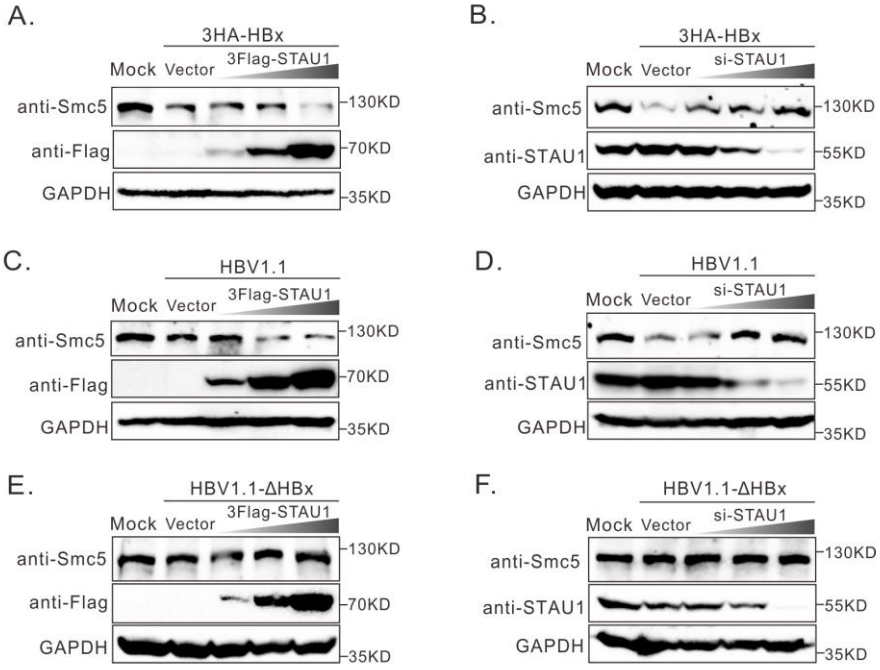

Figure S2. STAU1 regulates SMC5 levels via HBx, Related to Figure 8. (A) 3Flag-STAU1 overexpression decreased SMC5. Plasmid 3Flag-STAU1was transfected with plasmid 3HA-HBx into HepG2 cells. 48h later, western blot analysis was performed using SMC5 antibody. (B) STAU1 knockdown enhanced SMC5 levels. siRNA of STAU1 was transfected with plasmid 3HA-HBx into HepG2 cells. Western blot analysis was performed using SMC5 antibody. (C) to (F) STAU1 regulated SMC5 levels via HBx. HBV1.1 or HBV1.1-△HBx was co-transfected with 3Flag-STAU1 and siRNA against STAU1, respectively. 48h later, western blot analysis was performed using SMC5 antibody.
